# In Vitro Generation of Neural Cells with Regenerative Potential from Seabream Radial Glial Stem Cells: A Preliminary Study

**DOI:** 10.3390/biology15211941

**Published:** 2026-10-26

**Authors:** Maria Pia Santacroce, Adriana Lo Giudice, Valeria Buonfrate, Rosa Leone, Marco Cordisco, Claudia Castorani, Nicola Zizzo, Antonella Tinelli

**Affiliations:** 1 Department of Veterinary Medicine, University of Bari, 70010 Valenzano, Italy; adriana.logiudice@live.it (A.L.G.); v.buonfrate@phd.uniba.it (V.B.); rosa.leone@uniba.it (R.L.); marco.cordisco@uniba.it (M.C.); nicola.zizzo@uniba.it (N.Z.); antonella.tinelli@uniba.it (A.T.); 2 Institutional Affairs Directorate, University Language Centre, University of Bari, Multi-Functional Student Center, Piazza Cesare Battisti, 70121 Bari, Italy; claudia.castorani@uniba.it

**Keywords:** radial glia stem cells, neural stem progenitor cells, neuronal migration, nuclear translocation, neurogenic niche, teleost, gilthead seabream, primary neuroglial culture, CNS in vitro model

## Abstract

In marine teleosts, neurotoxicity and neuropathology research is currently constrained by a lack of validated in vitro models. Although zebrafish embryonic cell cultures provide a well-established platform for investigating the cellular and biochemical mechanisms governing neuronal growth and differentiation, comparable models for marine species remain sparse. To address this methodological gap, the present study establishes a primary neuroglial cell culture system from the brain of the gilthead seabream (*Sparus aurata*) to investigate central nervous system (CNS) neurogenesis by characterizing the morphological, immunophenotypic, proliferative, and migratory properties of radial glial (RG) cells and their neuronal progeny. This in vitro model likely recapitulates native CNS tissue architecture, enabling the detailed examination of neural stem cell cleavage modes, stem cell niche dynamics, and radial glial scaffolding. Our findings demonstrate that seabream RG cells may act as multipotent neural stem cells, generating young neurons through asymmetric division and directing their migration along radial glial fibers. Cells actively undergoing neurogenesis within the central zones of RG colonies exhibited co-localization of PCNA and UCHL1 immunoreactivity, confirming their active proliferative state. After 3 weeks in vitro, the central core of the colonies retained proliferative stem cell niches driving ongoing neurogenesis, whereas the peripheral regions comprised differentiated GFAP-positive astrocytes interwoven with extended neuronal processes. Furthermore, time-lapse imaging of nuclear kinetics provided direct visual confirmation of neuroblast locomotion along RG scaffolding, during which migrating neuroblasts exhibited sequential nuclear dilation and elongation as structural hallmarks of active movement. These in vitro proliferative dynamics closely mirror the permanent neural plasticity of the teleost brain, constituting a valuable tool for comparative neurobiology and neurotoxicology, as well as a potentially useful resource for models of central nervous system repair.

## Simple Summary

This study establishes an in vitro primary neuroglial culture system from *Sparus aurata* to model radial glia-mediated neurogenesis, demonstrating the capacity of radial glia to function as multipotent neural stem cells that drive neuronal differentiation and migration. Characterized through immunophenotypic profiling and time-lapse imaging, this cell culture model reveals active, continuous neurogenesis within proliferative UCHL1-positive central zones of radial glial colonies, effectively recapitulating the cellular plasticity observed in the in vivo teleost brain. Immunocytochemical analysis confirmed the establishment of a stable, highly viable primary in vitro neuroglial system. This central nervous system (CNS) model outlines the morphological and immunophenotypic profile of neuronal differentiation observed over gilthead seabream long-term culture. Overall, this standardized in vitro central nervous system (CNS) model provides a reliable experimental framework for future hypothesis-driven studies on teleost neurogenesis, functional CNS repair, neuropathological modeling, pharmacotoxicological screening, and neural tissue engineering.

## 1. Introduction

It was long believed that neurons and glial cells in the central nervous system arose from separate precursor populations that diverged early in development. This view stemmed in part from observations that neurogenesis and gliogenesis occur at different developmental stages, with neuronal production thought to end around the perinatal period. Previous studies indicate that, unlike mammals, teleost fish retain the vast majority of their radial glia (RG) throughout life, as observed in most freshwater species such as the *Oncorhynchus mykiss* [1]. Teleost RG display a characteristic morphology with somata predominantly localized in the periventricular zone. In species like the rainbow trout (*Oncorhynchus mykiss*), this spatial architecture supports the persistent neurogenesis and indeterminate brain growth typical of teleosts [1]. Whereas mammalian RG differentiate into astrocytes during development, rendering astrocytes the predominant mature glial population [2], the teleost brain maintains an extensive adult RG network [1]. For instance, in the hindbrain and spinal cord of the rainbow trout, the predominant cell population consists of GFAP-immunoreactive RG whose somata form a pseudostratified periventricular epithelium, giving rise to long radial processes that extend to the subpial surface and terminate in intensely immunostained endfeet [1]. This radial glial morphology is consistent with earlier descriptions in *Cyprinus carpio* [1,3]. Functionally, adult RG guide young migrating neurons from proliferative niches to their destinations, as demonstrated in *Apteronotus leptorhynchus* [4], and undergo asymmetric cell division to self-renew while generating neuronal progeny [5]. Consequently, the persistence of adult RG is strongly linked to continuous neurogenesis and robust regenerative capacity [6]. Although adult CNS neurogenesis occurs within dedicated niches across vertebrates, its efficiency varies widely [7,8,9]. Contrary to mammals, which possess limited regenerative scope, teleosts and amphibians reconstruct extensive CNS regions by enhancing neurogenesis and progenitor transdifferentiation [10,11,12]. However, the specific mechanisms governing RG-driven neurogenesis in marine species remain to be fully elucidated.

As vertebrates, zebrafish (*Danio rerio*) share extensive genetic and functional homology with humans, underscoring teleost models valuable for elucidating human neurobiological mechanisms [13]. In adult zebrafish, the premier in vivo fish model, brain lesions or complete spinal cord transection trigger robust neuroregeneration that functional motor recovery ensues. This repair is driven by resident neural stem cells (NSCs), multipotent radial glial progenitors capable of long-term self-renewal and differentiation into both neurons and glia [14]. Upon injury, these constitutive stem cells reactivate essential developmental signaling pathways to sustain ongoing neurogenesis. Conversely, mammalian CNS injuries exhibit restricted neurogenesis and insufficient axonal regrowth, failing to achieve complete functional repair [13].

In teleost models, adult neural stem progenitor cells (aNSPCs) persist lifelong, residing in various stem cell niches within the brain and spinal cord. Advanced studies have examined how various stimuli, such as neurotrauma, act on distinct populations of stem and progenitor cells, altering their cell cycle activity through the activation of normally quiescent aNSPCs or the modulation of niche-specific constitutive cell cycle rhythms [15]. The study of teleost neural stem progenitor cells (NSPCs) provides fundamental insights into the organization, differentiation, and proliferative dynamics of adult neurogenic niches [16]. Across various fish species, neural cell generation occurs throughout the lifespan [17]. Widespread proliferation zones across all subdivisions of the adult teleost rostrocaudal axis indicate ongoing adult neurogenesis in these brain regions [18,19]. For instance, adult zebrafish exhibit an extraordinary capacity for widespread neurogenesis throughout the central nervous system (CNS), with active proliferation zones located in the telencephalon, diencephalon, midbrain, cerebellum, and spinal cord [18,20]. In an in-depth study on zebrafish, Grandel et al. [20] identified approximately 16 distinct neurogenic niches along the ventricular surface, which harbor both self-renewing stem cell populations and mobile pools of neuronal precursors. Notably, Kaslin et al. [21] demonstrated that the adult zebrafish cerebellum contains bipotent radial glia-like stem cells capable of generating both neurons and glia for tissue homeostasis and repair. Following traumatic injury, radial glial cells act as neural stem cells by proliferating and giving rise to differentiated neurons that seamlessly integrate into pre-existing circuits. This extensive neurogenic capacity establishes the zebrafish as a premier model for studying neural repair, structural plasticity, and regenerative medicine [21]. Expanding our understanding of the niche-specific proliferative dynamics of adult neural stem and progenitor cells (aNSPCs) requires detailed investigation into the surrounding microenvironment and extracellular matrix components [22,23], which promises valuable insights for neural tissue repair [15]. A recent review by Caron et al. [15] underscores that adult zebrafish preserve lifelong developmental stem cell populations, including continuously dividing neuroepithelial cells (NECs) and quiescent or cycling radial glial cells (RGCs) [15]. Within stem cell niches, RGCs exist predominantly in quiescent or actively cycling states, whereas NECs undergo continuous proliferation [15]. Characterization of aNSPC kinetics in zebrafish typically relies on exogenous S-phase markers, such as the thymidine analogues BrdU and EdU [24], or the endogenous cell-cycle marker PCNA [15,25]. However, these approaches have rarely been applied to in vitro culture systems designed to mimic the native neurogenesis.

The scarcity of validated in vitro neural systems for Mediterranean teleosts has long constrained mechanistic research in marine neurotoxicity and neuropathology, necessitating reliance on whole-animal in vivo exposures or short-lived ex vivo brain slices. Consequently, despite the well-documented neurogenic potential and structural plasticity of the adult teleost brain, the cellular behaviors, morphological organization, and stem cell properties of radial glia-derived lineages in the gilthead seabream (*Sparus aurata*) remain poorly characterized under cell culture conditions.

Therefore, this study aimed to investigate radial glia-derived neurogenesis in a primary neuroglial cell culture model established from the brain of the teleost gilthead seabream (*Sparus aurata*). Although the freshwater zebrafish (*Danio rerio*) remains the benchmark teleost model for in vivo neuro-repair, its minimal brain mass severely constrains primary cell yields, presenting substantial technical limitations for complex downstream in vitro mechanistic assays. Conversely, the larger tissue biomass harvested per *S. aurata* brain facilitates the recovery of highly viable primary neuroglial suspensions that maintain structural and functional stability in long-term culture [25,26]. Despite these practical advantages, the specific neurogenic dynamics of *S. aurata* radial glia remain largely uncharacterized. Here, we systematically evaluated neural stem and progenitor cell (NSPC) dynamics, radial glial subpopulations, proliferative niche organization, and nuclear kinetics during neuroblast locomotion along elongated glial scaffolding. This reproducible primary culture system provides a robust experimental framework for comparative neurobiology, CNS repair modeling, and neurotoxicological research.

## 2. Materials and Methods

### 2.1. Animals

Juvenile gilthead seabream (*Sparus aurata*) with a mean body weight of 35 ± 5 g (*n* = 50) were obtained from a commercial hatchery (Panittica Pugliese, Brindisi, Italy). Fish were transported to the cell culture laboratory at the Department of Veterinary Medicine, University of Bari (Italy), in two 60-L oxygenated transport tanks filled with natural seawater (O_2_ at 10 mg/L, 37.5‰ salinity, 18 °C) at a density of 10 kg/m^3^. Upon arrival, fish were deeply anesthetized by immersion in seawater containing 0.02% (200 mg/L) 3-aminobenzoic acid ethyl ester (MS-222; Sigma-Aldrich, Milan, Italy). Anesthetized individuals were processed sequentially, wiped with 70% ethanol, and euthanized via spinal transection followed by decapitation. Only healthy individuals, as confirmed by anatomopathological examination, were selected for tissue sampling. Whole-brain tissues (~0.1–0.2 g/brain) were aseptically excised, pooled, and collected in sterile, pre-cooled Ca^2+^ and Mg^2+^ free Hank’s Balanced Salt Solution (HBSS; Sigma-Aldrich) supplemented with 10 mM HEPES, 0.5 mM EDTA, 25 mM NaHCO_3_, 200 IU/mL penicillin, 200 µg/mL streptomycin, 200 µg/mL amphotericin B, and 100 µg/mL gentamicin (Washing Solution, WS). Unless otherwise specified, all reagents and chemicals were purchased from Sigma-Aldrich (Milan, Italy).

### 2.2. Medium and Buffers

Phosphate buffered Saline 100 mM (PBS 10×, pH 7.4, autoclaved, 14.2 g/L Na_2_HPO_4_, 80.5 g/L NaCl, 2 g/L KCl), 1× PBS for cold washes and for the preparation of enzyme and antibiotic mixtures.

Hepes solution (pH 7–7.6, sterile filtered with Stericup 0.2 μm filter unit 500; Millipore, cat. no. SCGVU02RE,1 M-H0887, Sigma-Aldrich).

Washing buffer (pH 7–7.4, sterile cup filtered, HBSS; Sigma-Aldrich 250 mL/10 mM HEPES/0.5 mM EDTA/25 mM NaHCO_3_/2X antibiotic–antimycotic solution).

Digestion Medium (DM) (pH 7.2–7.4, sterilized by 0.22-µm filter unit, Leibovitz’s L-15 supplemented with 7 mM CaCl_2_/10 mM HEPES/25 mM NaHCO_3_/1× antibiotic–antimycotic solution).

Basal Medium (BM) (pH 7.2, sterilized by 0.22-µm filter unit, Leibovitz’s L-15 supplemented with 2 mM L-glutamine/10% FBS/1× antibiotic–antimycotic solution/1 mM sodium pyruvate/5 mM D-glucose/10 mM HEPES/12 mM NaHCO_3_/0.05% ITS+ (insulin/transferrin/sodium selenite/oleic acid/linoleic acid/BSA)/0.01 mM MEM non-essential amino acids/0.01 mM MEM vitamin mix/0.1 mM ascorbic acid/20 mM NaCl.

Enriched Differentiation Medium (pH 7.2, sterilized by 0.22-µm filter unit, L-15 BM supplemented with 10 ng/mL epidermal growth factor (EGF)/0.2 µg/mL hydrocortisone/10 mM glucose/10 µg/mL insulin/4 mM L-glutamine.

### 2.3. Isolation and Culture of Seabream Primary Neuroglial Cells

Primary neuroglial cultures were isolated from freshly excised brains under a vertical laminar flow microbiology safety hood, according to a recently developed method [27]. Briefly, cerebral hemispheres were excised using sterile surgical blades and scissors, and carefully cleared of meninges and blood vessels. After three washes with equal volumes of WS, brains were mechanically disrupted by pressing with a glass pestle through a 380-µm stainless-steel mesh filter. The resulting cell suspension was filtered and collected in enzyme-free Digestion Medium (DM), consisting of Leibovitz’s L-15 medium supplemented with 7 mM CaCl_2_, 200 IU/mL penicillin, 200 µg/mL streptomycin, 200 µg/mL amphotericin B, 100 µg/mL gentamicin, 10 mM HEPES, and 25 mM NaHCO_3_.

Enzymatic dissociation was performed by adding an enzyme cocktail containing 0.05% collagenase Type IV, 0.03% hyaluronidase Type IV-S, 0.3% dispase Type II, and 0.03% DNase Type I to the homogenate in DM for 20 min at 20 °C. Digestion was terminated by adding Leibovitz’s L-15 medium supplemented with 10% Fetal Bovine Serum (FBS; BioWhittaker, Lonza Walkersville Inc., Walkersville, MD, USA) to inhibit enzymatic activity. The mixture was sequentially filtered through 104-µm and 60-µm stainless-steel filters, the supernatant was discarded, and the pellet was resuspended in cold 1× phosphate-buffered saline (PBS) containing 5% fetal calf serum (FCS), followed by two centrifugations at 150× *g* for 10 min at 4 °C. The white cell pellet was recovered and incubated with 0.45 µg/mL DNase I in cold PBS; cell clumps were dissociated by gentle pipetting with a Pasteur pipette, followed by centrifugation at 70× *g* for 5 min at 4 °C. The final pellet was resuspended in L-15 medium containing 10% FBS and subjected to isopycnic separation via density gradient centrifugation to purify viable neuroglial cells from myelin debris, cellular fragments, and lighter, lipid-rich oligodendrocytes. The cell suspension, diluted 1:1 (*v*/*v*) with Leibovitz’s L-15 medium, was layered dropwise and gently onto a double density gradient (1:2 *v*/*v* cell suspension:Percoll) consisting of 15% and 60% Percoll (2:1 *v*/*v*) in 10× PBS. The 50 mL Falcon tubes containing the double gradient were prepared prior to isolation and maintained at 4 °C until use. Centrifugation was performed at 150× *g* (1400 rpm) for 10 min at 4 °C. Following centrifugation, a clear stratification of cell bands was visible: viable cells concentrated at the lower interface (forming a distinct white ring), whereas myelin debris and lighter, lipid-rich oligodendrocytes floated in the upper layers and were removed by aspiration. Using a fine-tipped Pasteur pipette, the white ring of viable cells was carefully collected, taking care to avoid the underlying erythrocyte pellet. The recovered cell suspension was washed in cold 1× PBS via two sequential centrifugation steps at 70× *g* (788 rpm) for 5 min at 4 °C. Finally, the cell pellet was resuspended in 3 mL of Leibovitz’s L-15 medium supplemented with 10% FBS, and the resulting suspension was subjected to cell counting. Cell viability was assessed via trypan blue exclusion. Brain tissue processing yielded 5.2 × 10^7^ viable cells/g tissue weight, with a mean viability of 92.8 ± 0.06%.

### 2.4. Seeding, Maintenance and Differentiation

The purified neuroglial cell suspension was adjusted to a density of 1 × 10^6^ cells/mL in Basal Medium (BM), consisting of Leibovitz’s L-15 supplemented with 2 mM L-glutamine, 10% FBS, 100 IU/mL penicillin, 100 µg/mL streptomycin, 100 µg/mL amphotericin B, 50 µg/mL gentamicin, 1 mM sodium pyruvate, 5 mM D-glucose, 10 mM HEPES, 12 mM NaHCO_3_, 0.05% ITS+ (insulin/transferrin/sodium selenite/oleic acid/linoleic acid/BSA), 0.01 mM MEM non-essential amino acids (MEM-NEAA; BioWhittaker), 0.01 mM MEM vitamin mix (BioWhittaker), and 0.1 mM ascorbic acid. BM osmolality was adjusted to seabream serum osmolarity (369 mOsm/L) by adding 20 mM NaCl as reported by other authors [27,28,29].

Cells were seeded at a density of 3 × 10^4^ cells/cm^2^ onto 0.01% collagen type I-coated substrates. For morphological characterization via phase-contrast microscopy, cells were seeded into 25-cm^2^ Falcon Primaria™ culture flasks (T25 flasks); for immunophenotyping via immunofluorescence, cells were seeded onto 12-well plates (Falcon, BD Biosciences; Franklin Lakes, NJ, USA). Cells were allowed to adhere for 12 h prior to replacing the culture medium with fresh BM, which was subsequently refreshed every 48 h. Cultures were maintained in a refrigerated incubator in a humidified atmosphere (97% air/3% CO_2_) at 18 °C (CO2-170 Innova; New Brunswick Scientific, PBI, Milan, Italy). During the second week of culture, to purify the neuroglial culture, microglia and oligodendrocytes were detached by shaking the T25 flasks on an orbital shaker. The initial agitation step was performed at 100 rpm for 1 h to detach microglia, which were subsequently discarded along with the supernatant. Following a brief wash with cold 1× PBS and the addition of fresh basal medium (BM), the T25 flasks underwent a second agitation step at 240 rpm for 6 h to release and remove oligodendrocytes. After a final wash with cold 1× PBS and the addition of Leibovitz’s L-15 Enriched Differentiation Medium (EDM), a purified layer of astrocytes and neurons remained adhered to the vessel surface. The absence of MBP (myelin basic protein) immunoreactivity confirmed that the resulting cell culture was devoid of oligodendrocytes.

Confluent monolayers were obtained after approximately 3–4 weeks. To establish neuroglial cell enriched cultures, cells were shifted after the 1 week to L-15 EDM, consisting of L-15 BM supplemented with 0.01 µg/mL epidermal growth factor (EGF), 0.2 µg/mL hydrocortisone, 10 mM glucose, 10 µg/mL insulin, and 4 mM L-glutamine. Adherent cells were monitored daily, and phase-contrast images were captured using a CCD camera mounted on a Motic AE31 inverted epi-fluorescence microscope (Motic, Milan, Italy). Given the low cellular yield per animal (0.1–0.2 g of brain tissue) and the laborious isolation procedure, brain tissue from all 50 animals was pooled into a single large-scale preparation to ensure a homogeneous cell population across all experimental groups. To ensure reproducibility, five independent seeding sessions were performed using T-25 flasks and ten sessions using 12-well plates (with six wells analyzed per antibody test). For each well, five microscopic fields were randomly selected and quantified. T-25 cultures were used to evaluate growth over time (up to 2 months) and for time-lapse imaging. Overall, a minimum of three independent tissue isolation procedures were conducted.

### 2.5. Antibodies

For indirect immunofluorescence, primary antibodies were diluted in PBS containing 3% BSA as follows: mouse anti-glial fibrillary acidic protein (GFAP) monoclonal antibody (mAb) (Sigma-Aldrich, Cat# G3893, clone G-A-5; 1:500); rabbit anti-ubiquitin C-terminal hydrolase L1 (UCHL1) polyclonal antibody (pAb) (Sigma-Aldrich, Cat# HPA005993; 1:1000); mouse anti-vimentin mAb (Sigma-Aldrich, Cat# V5255, clone VIM-13.2; 1:200); rabbit anti-neurofilament 200 (NF200) pAb (Sigma-Aldrich, Cat# N4142; 1:200); rabbit anti-microtubule-associated protein 2 (MAP2) mAb (Abcam, Cat# ab183830; 1:1000); mouse anti-neuronal nuclei (NeuN) mAb (Sigma-Aldrich, Cat# MAB377, clone A60; 1:100); rabbit anti-CD44 pAb (Sigma-Aldrich, Cat# HPA005785; 1:1000); rabbit anti-myelin basic protein (MBP) pAb (Sigma-Aldrich, Cat# M3821; 1:200); and mouse anti-nestin mAb (Abcam, Cat# ab22035, clone 10C2; 1:250). Secondary antibodies were diluted in PBS containing 3% BSA and applied for 45 min at room temperature (RT) as follows: TRITC-conjugated goat anti-mouse IgG (Sigma-Aldrich, Cat# T7028; 1:50); FITC-conjugated goat anti-rabbit IgG (Sigma-Aldrich, Cat# F6005; 1:50); FITC-conjugated goat anti-mouse IgG (Sigma-Aldrich, Cat# F0257; 1:50); and FITC-conjugated goat anti-rabbit IgG (Sigma-Aldrich, Cat# F9887; 1:50). For direct immunofluorescence, the following fluorochrome-conjugated primary antibodies were diluted in PBS/3% BSA and applied: FITC-conjugated mouse anti ß-III-tubulin mAb (Sigma-Aldrich, Cat# F2043, clone Tub 2.1; 1:100) and FITC-conjugated mouse anti-proliferating cell nuclear antigen (PCNA) mAb (Sigma-Aldrich, Cat# F0167, clone PC10; 1:100).

### 2.6. Microscopy Analysis

Brightfield and fluorescence imaging were performed using a Motic AE31 epi-fluorescent inverted microscope equipped with a DAPI/TRITC/FITC filter set (DAPI/Hoechst set: excitation D350/50×, emission D460/50m; FITC/RSGFP/Fluo-3/DiO/Acridine Orange set: excitation D480/30×, emission D535/40m; TRITC/DiI/Cy3 set: excitation D540/25×, emission D605/55m). Digital images were captured using a Moticam 3000C cooled CCD digital color camera (3.3 Megapixel, 1/2” CCD) controlled by Live Cam 1.0 and Motic Images Advanced (v3.2) acquisition software (Motic, Seneco, Milan, Italy). Image analysis, processing, and multi-channel overlay/merging were performed using Motic Images Advanced (v3.2) and Adobe Photoshop 8.0 (Adobe Systems Inc., San Jose, CA, USA). The images shown are representative of the consistent phenotypes observed across all 446 analyzed wells and microscopic fields.

### 2.7. Immunofluorescence

For indirect immunofluorescence, cells were rinsed three times with PBS, fixed with 4% paraformaldehyde (PFA) in PBS for 15 min at room temperature (RT), washed twice with PBS, and permeabilized with 0.2% Triton X-100 in PBS for 10 min at RT. Following three 5-min washes with PBS, non-specific binding sites were blocked by incubating cells in PBS containing 3% BSA (blocking buffer) for 30 min at RT. Cells were then incubated with primary antibodies diluted in blocking buffer for 60 min at RT. After three washes with PBS, cells were incubated with the corresponding fluorophore-conjugated secondary antibodies for 60 min at RT in the dark. Following three additional PBS washes, cell nuclei were counterstained with 4′,6-diamidino-2-phenylindole (DAPI; 1.5 µg/mL) for 10 min at RT. To mitigate photobleaching, stained cells were mounted in an antifade mounting medium composed of 90% glycerol and 100 mg/mL 1,4-diazabicyclo [2.2.2]octane (DABCO) in PBS, which was applied directly to the wells prior to microscopic examination. Negative controls were processed following the exact same protocol, omitting the primary antibody incubation step.

### 2.8. Live Cell Imaging

Live-cell imaging was used to evaluate the cellular dynamics of *Sparus aurata* neuroglial cultures in 2D. Cells were seeded at a density of 3 × 10^4^ cells/cm^2^ onto 0.01% collagen type I-coated 25-cm^2^ Falcon Primaria™ culture flasks with plug-seal caps (T-25 Falcon Primaria™, BD Biosciences; Sismed s.r.l., Bari, Italy) containing Leibovitz’s L-15 BM. To permit gas exchange, flasks were incubated with slightly loosened caps at 18 °C in a refrigerated incubator with a humidified atmosphere of 97% air/3% CO_2_ (CO2-170 Innova, New Brunswick Scientific, PBI, Milan, Italy). On day 10 of culture, flasks were tightly closed, sealed with Parafilm, and incu-bated overnight at 18 °C under CO_2_ free conditions in a temperature-controlled chamber. Leibovitz’s L-15 medium is specifically formulated for CO_2_ free or low CO_2_ systems via phosphate and amino acid-based buffering system rather than a sodium bicarbonate system, rendering it suitable for cultures where CO_2_ usage is temporarily restricted. Live imaging of cell dynamics was then monitored over a 12-h period. The imaging system comprised a Motic AE31 epi-fluorescent inverted microscope equipped with a Moticam 3000C Cooled CCD digital color camera (3.3 Megapixel, 1/2″ CCD), controlled by Motic Images Advanced (v. 3.2) acquisition software with Live Cam 1.0 (32-bit) (Motic, Seneco, Milan, Italy). The T-25 flask was secured under a 20× objective within the temperature-controlled chamber maintained at 18 °C. Time-lapse images were automatically captured every 15 min for 12 h. Image processing and video sequence assembly were performed using Motic Images Advanced (v3.2).

### 2.9. Quantification of Migration Modes and Mitotic Somal Translocation (MST)

The migration trajectory of individual cells was automatically tracked over time at 15-min intervals. Cells were classified as undergoing radial migration if the angle be-tween their primary vector of displacement and the radial glial scaffold was between 0° and 30°. Conversely, cells were classified as undergoing tangential migration if their trajectory was orthogonal to the glial scaffold, maintaining an angle between 60° and 90° relative to the radial axis, while exhibiting a characteristic multipolar or branched morphology.

Mitotic somal translocation (MST), a specialized dynamic behavior of cycling radial glial progenitor cells, was distinguished from standard interphase somal translocation based on the criteria established by Wang et al. [30] and Ostrem et al. [31]. MST was defined as a rapid, directed soma translocation along the primary basal process covering a distance >20 µm (approximately one cell diameter) at a velocity of >20 µm/h within a short time window (<60 min) immediately preceding cytokinesis. This translocation event culminated in cell rounding, apical-to-basal nuclear displacement, and horizontal cleavage division.

## 3. Results

### 3.1. Morphological Characterization of Neuroglial Cell Types by Phase-Contrast Microscopy

Firstly, we evaluated the development and differentiation of neuronal lineages from radial glial stem cells (RGSCs) in gilthead seabream (*Sparus aurata*) primary cultures using direct and inverted phase-contrast microscopy. Morphological analysis revealed that 24 h after plating, freshly isolated neural stem cells (NSCs) displayed a distinctive ovoid shape (Figure 1A). By day 2, early elongated, adherent RG stem progenitors (RGSPs) exhibited initial proliferative activity along their cellular extensions (Figure 1B,C,E,F,J). As shown in Figure 2 and Figure 3, phase-contrast microscopy of *Sparus aurata* RGSPs identified the emergence sites of initial polar protrusions within the first few days in vitro. Over the first 2 weeks of culture, microscopic examination confirmed three distinct cell populations: radial glia stem progenitors (RGSPs), which formed clusters with high proliferative capacity and an irregular, polygonal morphology (Figure 1B,C,E); neural stem cells (NSCs), characterized by an ovoid shape at day 1 that progressed to a bipolar or tripolar elongated morphology from day 2 (Figure 1D,I) to day 6 (Figure 2A,B); and neural progenitor cells (NPCs), which committed to neuronal lineages without generating glia (Figure 1F–H).

### 3.2. Radial Glial Colony Formation and Proliferative Niches

Representative images of newly formed and actively dividing RGSP colonies are presented in Figure 2A,B,D and Figure 3. Within 1 week of plating, radial progenitor cells extended their processes radially around the colony periphery, acquiring the characteristic morphology of mature RG cells (Figure 2D and Figure 3A). High-magnification microscopic analysis of these multipotent RGSP colonies revealed prominent growth cones at the emergence sites of radial processes, representing the early stages of glial fiber development (Figure 2C,C’). As RG progenitors initiated process extension, they formed basal cones that generated long, directional processes while simultaneously emitting small lateral extensions for substrate attachment and intercellular interaction (Figure 2B). Within the colony, these early basal cells (bRGCs) divided rapidly to form clusters of newly generated post-mitotic cells, establishing distinct microenvironments that function as actively proliferating stem cell niches (Figure 2D and Figure 3A–C).

During the proliferation of basal radial glial (bRG) cells via symmetric division, which expands the parental progenitor pool, the nucleus of the newly generated daughter cell distal to the basal origin is displaced longitudinally into the growing apical extension (Figure 2D, yellow dashed outline). The basal portion of the glial cone subsequently extends via an elongated process toward the distal tip, giving rise to new RG progenitors (Figure 3D–F). Conversely, during asymmetric division, the daughter cell distal to the proliferating base is smaller and becomes basally displaced relative to the mother cell, subsequently giving rise to a neuroblast during neurogenesis (Figure 3F).

### 3.3. Mitotic Cleavage Dynamics of Radial Glial Progenitor Cells

During the early stages of in vitro neurogenesis, radial glial progenitor cells (RGPs) undergo asymmetric divisions to generate a new apical progenitor alongside a basal progenitor, with the latter driving expansion of the radial colony (Figure 2D). Most basal progenitor divisions are vertically oriented relative to the colony axis, thereby promoting self-renewal. Cleavage plane alignment demonstrates that vertical cleavage planes typically result in symmetric division, whereas horizontal or oblique cleavage planes frequently trigger asymmetric division, likely due to the unequal inheritance of polarized apical and basal cellular components. The transition from a proliferative to a neurogenic state gives rise to the primary parental progenitors that initiate neurogenesis: radial glial stem cells (RGSCs). Notably, the division patterns of RGP cells shift predictably across distinct culture stages.

Specifically, symmetric proliferative division predominates during early development, wherein RGP cells divide symmetrically to yield two identical daughter cells, thereby expanding the initial progenitor pool (Figure 2 and Figure 3). Asymmetric neurogenic division becomes prevalent during intermediate differentiation stages; during this phase, one daughter cell retains RGP identity to maintain the progenitor pool, while the other differentiates into a committed neuron or an intermediate progenitor cell (IPC) (Figure 2D). Finally, late stage development is characterized by symmetric gliogenic division, a terminal symmetric division in which both daughter cells commit to glial lineages, such as astrocytes, ultimately depleting the progenitor pool.

### 3.4. Neural Stem Cell Self-Renewal Pathways

Neural stem cell (NSC) division utilizes tightly regulated mitotic mechanisms to maintain the long-term progenitor pool through three distinct pathways. Symmetric self-renewal involves an NSC dividing into two identical multipotent NSCs to expand or replenish the stem cell reservoir (Figure 1D,I and Figure 2D). Asymmetric self-renewal occurs when an NSC yields one self-renewing NSC and one committed progenitor cell, a process that operates continuously within mature neurogenic niches. Alternatively, this division mode can directly generate small neural progenitor cells along the soma or within bipolar and tripolar processes (Figure 1). When basal radial glial cells (bRGCs) proliferate via symmetric division, the nucleus of the daughter cell distal to the basal origin translocates immediately following mitosis. This longitudinal displacement propels the nucleus into the growing distal extension (Figure 3D).

This longitudinal displacement occurs during the expansion of the parental progenitor pool; these frequent symmetric divisions within the bRGC colony are demarcated by yellow dashed outlines in Figure 2. Conversely, during asymmetric bRGC division, the newly generated daughter cell distal to the proliferating base is smaller and basally displaced relative to the mother cell, subsequently giving rise to a migrating neuroblast (Figure 2). Early-stage neurogenesis initiates with the proliferation of these neural progenitor cells, where highly abundant basal radial glial progenitors (bRGPs) drive final neuronal output (Figure 4A), followed by functional maturation of neurons and glia in subsequent stages.

Morphological analyses of *S. aurata* primary radial glia in vitro reveal a reversal of the classic in vivo apicobasal polarity axis. In classical cortical neuroanatomy, the developmental axis is defined as apicobasal, extending from the ventricular zone (apical) to the pial surface (basal). Conversely, in *S. aurata* neuroglial cultures, the developmental axis originating from the colony core follows a basal-to-apical (distal) orientation (Figure 3 and Figure 4C), extending outward from the adherent bRGC parental colony base toward the upper radial fiber scaffold.

### 3.5. Mechanisms of Radial and Tangential Migration

The primary distinction between radial and tangential migration lies in the structural scaffold utilized and the degree of nuclear deformation required during nucleokinesis (Figure 4A–C and Figure 5). During radial migration, neurons utilize radial glial fibers as a physical scaffold, which consists of long glial processes spanning the entire thickness of the monolayer (Figure 4A,B). The neuron adheres to and enwraps the glial fiber via fibrous extensions (Figure 5). Throughout this process, nucleokinesis is strictly guided; the neuronal nucleus remains aligned with the fiber and undergoes continuous longitudinal elongation to advance along this narrow track (Figure 4A,B). Concurrently, cell adhesion sites maintain extensive contact with the glial scaffold (Figure 5F). Conversely, tangential migration does not rely on a perpendicular scaffold; instead, neurons move transversely parallel to the substrate (Figure 3 and Figure 4C). In this configuration, the migratory scaffold is more dynamic, and neurons frequently utilize pre-existing axons as tracks, interlace with one another, or migrate transversely by transitioning between adjacent fibers along the periphery of the radial glial cell (RGC) colony (Figure 3A,B,C,F). This structural arrangement grants the cells greater directional freedom to traverse extensive distances (Figure 3B,F and Figure 4C). Here, nucleokinesis is characterized by saltatory movements, and the nucleus undergoes irregular morphological changes because it is not mechanically constrained by a single glial fiber (Figure 4C). Consequently, locomotion relies heavily on the dynamic exploration of the microenvironment by the leading process (Figure 4C).

### 3.6. Cytoskeletal Dynamics and Mitotic Somal Translocation

Radial migration predominates during the first 3 weeks of culture. Specifically, neuroblasts migrate along radial glial cell processes, which serve as directional tracks. The migrating cell adheres to the glial fiber via specialized adhesion mechanisms and translocates along its outer surface toward the distal tip (Figure 4A,B and Figure 5). At this site, mitotic division initiates, generating neuronal progeny anchored to the fiber tip (Figure 4A,B). Upon completion of cytokinesis, the neuroblast resumes migration in the opposite direction, translocating proximally along the fiber, while the newly generated, small neuronal cell concurrently extends adhesive processes around the fiber shaft (Figure 4A,B). This rapid, terminal shift to the division site is driven by mitotic somal translocation (MST), as revealed by real-time imaging of a bRGPC undergoing MST in a 2 week-old culture (Figure 4A,B). In migrating neurons, the centrosome is consistently positioned ahead of the nucleus, oriented toward the leading process. This anterior localization of the centrosome confirms that the enrichment zone of contractile proteins (the cytoplasmic bulge) is situated strictly on the opposite, posterior side of the cell during forward somal translocation (Figure 4B). Furthermore, newly generated neuroblasts utilize these fibers as tracks to undergo gliophilic migration. Through nucleokinesis, a highly coordinated two-step mechanism, the neuron adheres to the glial fiber and translocates from its site of origin toward the expanding fiber scaffold or the injury site (Figure 4B). The coordination of nucleokinesis during cellular scaffolding is a dynamic process that enables the nucleus to advance along a structural track by modulating its morphology. In this context, nuclear elongation is not a passive phenomenon but a critical mechanical adaptation required to navigate dense or confined environments (Figure 4C). Nucleokinesis generally occurs in two alternating phases coordinated by cytoskeletal remodeling (Figure 4B). The initial phase involves protrusion, during which the centrosome and the Golgi apparatus migrate forward into a cytoplasmic dilation within the leading process (Figure 4B). This phase is characterized by neurite elongation and the orientation of the centrosome toward this cytoplasmic bulge (Figure 4B). The second phase consists of nuclear translocation, where the nucleus advances toward the previously displaced organelles. This movement is driven by contractile proteins accumulated at the cell rear, which generate the mechanical forces required to propel the nucleus forward (Figure 4B). During migration back to their site of origin, neurons extend a leading process and form a cytoplasmic dilation within its proximal region, into which the elongating nucleus translocates. Conversely, within the long processes of radial glial progenitor stem cells, a cytoplasmic swelling forms in a relatively distal region, followed by nuclear entry. Concurrently, the newborn daughter cell begins to establish adhesions and extend processes around the parental glial fiber, thereby facilitating the expansion of the cellular scaffold. Time-lapse images reveal that the cell soma exhibits a dynamic, comet-like behavior characteristic of migrating cells (Figure 4A,B).

### 3.7. Long-Term Neuronal Maturation and Astrocytic Scaffolding

During neural development, neural progenitors proliferate to generate immature neuroblasts, which undergo sequential morphological transitions as they migrate radially along radial glial processes (Figure 3E,F and Figure 4C). Upon initiating migration along the internal culture scaffold, newly formed neurons enter a multipolar phase. Crucial maturation events occur during this stage, including the establishment of neuronal polarity and axonal outgrowth (Figure 6 and Figure 7). Many primary neurons begin emitting neurites within 4–6 days post plating, with extensive branching completed after 2 weeks in culture (Figure 5 and Figure 6).

Subsequently, these cells assume a bipolar morphology characterized by an anterior process that develops into a putative apical dendrite (Figure 6). During neuritogenesis, neurons emit neurites that grow increasingly larger over time, completely covering the culture with filamentous processes (Figure 7). When maintained in enriched medium, primary neuroglial cultures initially differentiate into a mature monolayer of stellate astrocytes (Figure 6). Following 2 weeks of culture, long, branched neurites become visible (Figure 6A,B), and this astrocytic monolayer begins to actively generate neurons (Figure 3 and Figure 6). Within this microenvironment, astrocytes provide critical migratory cues and structural scaffolding support (Figure 6A). Concurrently, sequential neurogenesis proceeds; RGPs generate early-born, deep-layer-like neurons that remain intimately intertwined with the glial fiber scaffold, before transitioning to produce later-born, upper-layer-like neurons that overlie the astrocytic monolayer (Figure 7). This sequential production establishes a cooperative scaffold composed of radial glia and astrocytes that promotes collective neuronal migration.

During terminal maturation, late differentiation of RGCs into astrocytes occurs via gliogenesis (Figure 5 and Figure 6). By the fourth week of culture, the astrocytic monolayer is densely covered by neuritic-like and dendritic-like branching, alongside well-defined neuronal-like morphology (Figure 7B). Neurite-like arborization and length progressively increase over time, peaking at 2 months in vitro (Figure 7B–C’).

### 3.8. Phenotypic Characterization and Marker Expression

We characterized the temporal dynamics and expression profiles of neuroglial markers via immunofluorescence microscopy. Following three weeks in vitro, radial glial cells (RGCs) displayed marked immunopositivity for both glial fibrillary acidic protein (GFAP) and vimentin (Figure 8A). Proliferating colonies exhibited prominent nestin expression, particularly within the processes of intermediate progenitor cells (IPCs) in 1-month-old colonies, confirming a radial glia-like progenitor phenotype (Figure 8B).

Differentiated astrocytes expressed both CD44+ and GFAP+ (Figure 8H). Those located at the outer periphery of RGC colonies possessed elongated processes aligned with corresponding βIII-tubulin+ neuronal fibers, eventually acquiring a classic stellate morphology upon maturation (Figure 8E–G). In contrast, the inner zone retained specialized niches of mitotically active glial stem-like cells that sustained robust neuronal production throughout long-term culture. Regarding the neuronal lineage, β-III-tubulin effectively labeled microtubules, somata, and neurites across both immature and mature neurons, providing a reliable marker to track early neurogenesis within RGC colonies (Figure 8E,G). Figure 8 and Figure 9 demonstrate that during neurogenesis, cells undergo a definitive shift in their molecular marker profile as they transition from dividing progenitors to post-mitotic, mature neurons, progressively acquiring a mature phenotype.

Whereas NeuN expression was absent in actively mitotic cells (Figure 9L,L’) after one month in vitro, differentiating neurons exhibited a high density of neurofilament-positive (NF^+^; Figure 8) and MAP2-positive (MAP2^+^; Figure 9) processes, with β-III-tubulin^+^ neuronal fibers running alongside and intermingling with the elongated processes of GFAP^+^ astrocytes. However, after 2 months in culture, actively replicating neuronal progenitors (UCHL1^+^/PCNA^+^) were clearly visible within the center of a GFAP^+^ radial glial colony, suggesting the maintenance of a stem/progenitor phenotype (Figure 9A–D). After 2 months in culture, strong UCHL1 immunoreactivity indicated ongoing neurogenesis, characterized by newly generated neurons migrating along established radial fibers (Figure 9C,D), thereby confirming the production of neuronal progeny. Neural progenitor cells were NeuN^−^/PCNA^+^, indicating their proliferative capacity. As neuroblasts differentiate, they exit the cell cycle, downregulating PCNA expression (PCNA^−^), and enter a permanent post-mitotic state while simultaneously upregulating NeuN, confirming their commitment to a mature neuronal lineage. This gradual process is clearly illustrated in Figure 9L,L’, where differentiating neurons (NeuN^+^) progressively cease dividing (PCNA^−^), slowly reversing the PCNA marking, and acquire the marker profile of post-mitotic neurons (NeuN^+^), surrounded by actively proliferating progenitor cells (NeuN^−^/PCNA^+^).

The images in Figure 10 illustrate the expression of specific markers via double immunofluorescence showing tangential and radial neuronal migration, as well as structural interactions along radial glial cell fibers. After 2 months in vitro, β-III-tubulin^+^ neurons (Figure 10A,A’; green) appear aligned with a radial glial fiber (GFAP^+^; red). While the neuronal soma displays a prominent cytoplasmic protrusion (green), the β-III-tubulin signal highlights the perinuclear ring and the centrosome/microtubule-organizing center (MTOC), indicating phases preparatory to nuclear translocation and cell migration as reported by Powers et al. [32]. Punctate β-III-tubulin^+^ foci, corresponding to the centrosome and MTOC, migrated in tandem toward the leading process as positive signals invaded the growth cone filopodia (Figure 10). Such dynamic behavior aligns with previous observations from time-lapse microscopy, where saltatory or comet-like soma movement was characteristic of actively migrating neurons. Concurrently, real-time imaging demonstrated dynamic, transient extension and retraction of membrane protrusions (or neuritic varicosities) along motile segments of the neural fibers (Figure 4A,B). During tangential and radial migration, a characteristic nuclear orientation is observed; specifically, elongated nuclei are aligned along the axis of the extending neurites (Figure 10B–D; green). Along migration and nuclear translocation, nuclear elongation is accompanied by asymmetric cell division of precursor cells (Figure 10B,C’). Visualization of GFAP^+^ regions (Figure 10C,C’), combined with UCHL1^+^ neuronal clusters (Figure 10D) and PCNA^+^/DAPI^+^ nuclei (Figure 10B,C’,D), reveals the proliferative capacity of cells assembled within the radial glial scaffold. High-contrast, inverted grayscale images highlight the complex structural organization and branched processes of the scaffold guiding migration. Figure 10D shows specific physical contacts that facilitate the adhesion of neuroblasts to radial glial processes along structural radial fibers during nuclear elongation.

### 3.9. Neural Niche Architecture and RGC Colonies

Neural stem cell colonies established a specialized niche for radial glial cells (RGCs), where cell polarity and anchoring relied on cell–cell adhesion (Figure 5E,F). In Figure 10D, white arrowheads highlight initial adhesion filaments extending from neural cells at the onset of migration, as the nucleus undergoes elongation (Figure 10B,C’). This localized expression suggests that N-cadherin-mediated interactions facilitate the adhesion of RGCs and migrating neurons to structural radial fibers, thereby maintaining the architectural integrity of the neurogenic niche.

Radial glial cell (RGC) colonies exhibited a precise spatial segregation of distinct cell types. Staining for PCNA, a marker of DNA synthesis during the G1/S phase, revealed ongoing replication localized to the central core, in contrast to the mature peripheral zones (Figure 9). Within the first week of culture, early-replicating cells in the central core began differentiating into committed neurons along the colony margin, reaching full maturation within a timeframe ranging from 2 weeks to 2 months in vitro (Figure 6 and Figure 9). Double immunofluorescence demonstrated that the outer rim consisted primarily of differentiated GFAP^+^ astrocytes, closely intermingled with neuronal processes. Conversely, the inner zone harbored clones of mitotically active RGCs (DAPI^+^/PCNA^+^). These central aggregates constituted highly active neurogenic sites, where UCHL1 immunoreactivity revealed newly generated neurons migrating along pre-existing radial fibers (Figure 9C–H). Counterstaining confirmed that these emerging neuronal cells, positive for MAP2, derived directly from the proliferating progenitor population (Figure 9). To distinguish between the two distinct radial glia populations and the coexisting, clonally related glial and neuronal cells, GFAP labeling was combined with UCHL1 labeling. GFAP^+^ regions were UCHL1-negative, whereas the newly formed neuronal aggregates were UCHL1^+^ and PCNA^+^/DAPI^+^, strongly suggesting that these actively proliferating precursor cells originated from radial glia (Figure 9). At various stages following lineage commitment, neuroblasts cease dividing (rather than continuing to expand the precursor pool), aligning their elongated (DAPI^+^) nuclei along short β-III-tubulin^+^ neurites. This highlights the evolution of arborization with intersecting branches during the phases of tangential and radial migration along the neuritic tree (Figure 10).

β-III-tubulin serves as a benchmark pan-neuronal marker, as this cytoskeletal protein is expressed almost exclusively in both developing and mature neurons, making it ideal for visualizing neuronal cell bodies, axons, and dendrites. In Figure 10A,A’, neurons labeled with β-III-tubulin–FITC display a cytoplasmic swelling (green bulge) adjacent to the nucleus along with punctate, bead-like markings along the fiber. Single dots containing both the centrosome and the microtubule-organizing center (MTOC), positive for beta-III tubulin, migrate in concert, while the positive dots invade the filopodia of the growth cone (Figure 10A). Morphologically, this profile represents neurons undergoing development, preparing for migration, or undergoing regeneration; specifically, the staining highlights the perinuclear ring, the centrosome/microtubule-organizing center (MTOC), or an early growth cone [32]. By 1 month in culture, the cells formed a confluent monolayer of polygonal–stellate astrocytes that were Vimentin^−^ but strongly immunoreactive for CD44 and GFAP (CD44^+^/GFAP^+^).

## 4. Discussion

The modes of neuronal migration along the primary radial glial scaffold in marine teleost radial glial cells (RGCs), as well as their regenerative potential, remain poorly understood due to a paucity of stable in vitro systems. Findings regarding the neurogenic zones of the *Sparus aurata* brain remain scarce. To date, only Zikopoulos et al. [33] have investigated its in vivo plasticity, utilizing 5-bromo-2′-deoxyuridine (BrdU) immunocytochemistry to map proliferation zones across different life stages and sexual phases. Their analysis of adult male and female brains revealed ongoing adult neurogenesis within ventricular and subventricular regions, primarily corresponding to embryonic proliferative niches. The highest mitotic activity was identified in the molecular layer of the corpus and valvula cerebelli, with significant proliferation also observed in hypothalamic, thalamic, and telencephalic ventricular zones, as well as along the margins of the optic tectum. This proliferative pattern, characterized by a higher density of newly generated cells in females, supports the hypothesis of continuous neurogenesis in the adult teleost brain [33]. Our study demonstrates that radial glial cells (RGCs) serve as the primary adult neural stem cells (NSCs) responsible for continuous neurogenesis in the *Sparus aurata* brain cultures.

We observed that NeuN+ cells constituted 15% of the population in 3–4-day cultures (compared to Nestin+ cells, which accounted for 85%), rising to 45% at 2 weeks and stabilizing at 30% between the third and fourth weeks. Given that the NeuN+ population in early-stage cultures is markedly lower than in later stages (2 and 3–4 weeks), it can be inferred that the culture undergoes a significant temporal enrichment of the neuronal lineage; this is consistent with the active progression of the progenitor lineage rather than the static maintenance of neurons in vitro. Early-stage cultures (days 3–4) showed active synthesis of both neurofilaments (NF, 23%) and glial fibrillary acidic protein (GFAP, 18%). This early NF expression begins in neuroblasts that have just committed to differentiation, prior to complete nuclear maturation of NeuN. Meanwhile, GFAP is expressed not only by differentiated astrocytes but also by RGPCs and the RGCs themselves. The delayed appearance of NeuN+ cells relative to Nestin+ cells indicates an initial predominance of neural stem/progenitor cells over mature neurons. Over the 3–4 week culture period, the progressive increase in NeuN+, GFAP+ (up to 49%), and NF+ (up to 78%) cells demonstrates the active differentiation of progenitors into mature neural lineages, giving rise to new mature neurons in vitro. NeuN expression naturally increases as post-mitotic neuroblasts undergo nuclear maturation; conversely, had mature neurons simply survived the initial tissue dissociation, their proportion would have steadily declined. Concurrently, astrocytes differentiate alongside neurons from bipotent or multipotent neural progenitor cells (NPCs). In the absence of microglia or oligodendrocytes, the glial population consists almost entirely of GFAP-positive astrocytes and RGCs.

Previously, we demonstrated that *S. aurata* brain cultures harbor radial glia displaying a distinct morphology, a characteristic molecular signature, and notable proliferative capacity [26,28]. These findings prompted us to further investigate nuclear translocation and migratory behavior during neurogenesis, as well as glial lineage progression and neuroblast differentiation. Using this in vitro model, we identified basal radial glial cells (bRGCs) as multipotent, self-renewing progenitors capable of generating both neurons and astrocytes (Figure 11).

Furthermore, as previously demonstrated in mammals [34,35], these cells exhibit mitotic somatic translocation (MST) and interkinetic nuclear migration (IKNM), underscoring their utility as a comparative model for central nervous system (CNS) neuronal migration during tissue regeneration. Unlike the mammalian CNS, where radial glial cells are transient embryonic structures, radial glia represent the predominant neuroglial population in the adult teleost CNS [36]. Morphologically, however, teleost RGCs closely resemble their mammalian counterparts, displaying long radial processes that are immunopositive for Vimentin and GFAP, as our findings demonstrate. In the mammalian neocortex, these embryonic progenitors generate both neurons and glia, with Noctor et al. [37] definitively establishing that proliferating radial glia directly give rise to neurons. These progenitors maintain mitotic activity throughout neurogenesis before differentiating into astrocytes post-migration [37]. Parallel to these mammalian mechanisms, our findings confirm that teleost neurons migrate along progenitor radial extensions toward their functional destinations, confirming that the fundamental role of radial glia in choreographing neuronal migration is evolutionarily conserved [38].

During neurogenesis, radial glial fibers regulate the direction of nucleokinesis within extending processes, highlighting that the centrosome, or microtubule-organizing center (MTOC), serves as a key indicator of cell polarity. Recent studies have demonstrated that centrosomal positioning is precisely regulated during neurogenesis, migration, and differentiation, enabling efficient nucleokinesis and directional neurite outgrowth [39]. Several investigations converge in stating that this process requires cytoplasmic dynein, cell polarity proteins, and microtubule-associated proteins that coordinate microtubule remodeling [40]. These proteins first coordinate the positioning of the MTOC in the anterior process ahead of the nucleus, subsequently driving the movement of the nucleus toward the centrosome. A similar finding was previously reported in mouse RG-like cells undergoing mitotic somal translocation, a process in which centrosome movement within the basal process during interphase precedes nuclear translocation [30]. Consistent with our findings, centrosomal positioning, alongside the dynamic regulation that couples and uncouples the nucleus, underlies the directional migration of neurons [40]. Centrosome behavior governs the neuron’s mode of migratory advance. During classic two-phase saltatory movement, the centrosome first advances into a swelling in the leading process; subsequently, the nucleus translocates toward the centrosome via a forward leap, completing the step [39].

Moreover, our findings demonstrate that radial glia not only generate nascent neurons but also maintain or reconstruct the scaffold required for their migration. This scaffold self-renewal occurs because RG undergo symmetric divisions to produce new glial cells that extend long cytoplasmic processes. These fibers span the entire thickness of the culture, establishing a structural network that provides critical physical guidance. In 3- to 4-week-old cultures, an intricate network of dense glial processes integrated within neural networks becomes evident. Based on immunocytochemistry results evaluating cell culture composition over time, cells from purified neuroglial cultures were demonstrated to synthesize proteins recognized by neurofilament (NF) and GFAP antibodies after 3–4 weeks in culture. This confirms the presence of a mixed population of functionally active neurons and astrocytes capable of long-term survival and growth. By week 4, this structural maturation was evidenced by extensive neurite elongation and dense axonal arborization overlying the underlying astrocytic layer (Figure 7). Pre-coating culture plates and flasks with 0.01% type I collagen was crucial for maintaining long-term viability and structural complexity by enhancing cell adhesion stability and facilitating cell–cell interactions during network assembly. Additionally, enriched differentiation medium (EDM) proved essential for preserving precursors in a stem-like proliferative state while providing the metabolic support required for neural differentiation.

Further analysis revealed that within 2-month-old long-term cultures, prominent fibrils are interspersed between neuronal and glial cell bodies, establishing complex structural connections between glial filopodia and axonal branches. Neuronal migration involves extensive morphological remodeling characterized by sequential nuclear dilation and elongation. Long-term neuroregeneration depends on coordinated neural stem/progenitor cell (NSPC) proliferation, migration, differentiation, survival, and network integration. Here, the dynamic network of radial glial fibers forms a flexible structural scaffold that drives neuronal growth and migration, as confirmed by the presence of dividing nuclei along these processes. Elucidating these cellular and molecular mechanisms may reveal signaling pathways to modulate neural remodeling through neurogenesis and gliogenesis, or to accelerate the differentiation of newborn neurons.

Over the past decade, studies on the adult zebrafish have highlighted the contribution of quiescent radial glial cells (RGCs) to proliferation following brain injury. Following optic tectum injury, RGCs possess the capacity to generate newly generated neurons [41,42]. Such in vivo studies conducted in zebrafish demonstrate that the neuronal regeneration process is driven by a combination of reactivated RGCs and constitutively proliferating neuroepithelial cells (NECs) [15]. In young fish, RGCs play a major role in the regenerative process alongside NECs, giving rise to neuronal phenotypes similar to those produced during constitutive neurogenesis [15]. To enhance repair, regenerative strategies could target neural stem/progenitor cell (NSPC) migration, differentiation, and lesion-site survival by modulating the neurogenic niche via growth factor stimulation. Because gilthead seabream basal radial glia (bRGs/bRGCs) exhibit highly efficient neurogenic capacity, they offer a powerful model to accelerate damage repair mechanisms. Consequently, this model of regenerative neurogenesis is particularly well-suited for the in vitro screening of cell therapies, neurotrophic factors, and pharmacological agents aimed at enhancing the regenerative capacity of neural stem cells.

Immunocytochemical analysis using DAPI and PCNA identified distinct proliferative subpopulations among NSPCs in newly seeded and long-term neural cultures. In this teleost model, molecular markers of adult-like glial precursors, such as Vimentin and GFAP, exhibit high specificity in adult NSPCs. Crucially, basal radial glia (bRGCs) exhibited long-term maintenance of their neurogenic capacity. Within these long-term cultures, specialized neurogenic niches maintain cells in an undifferentiated state, ensuring the spatiotemporal generation of mature phenotypes within a continuously evolving developmental context. Furthermore, the specific expression patterns and subcellular localization of vimentin and GFAP observed in these marine cultures facilitate the phenotypic characterization and cell-type identification of radial glial cells. Previous studies on the characterization of an adult tilapia (*Oreochromis* sp.) neural progenitor cell line demonstrated that these cells co-express GFAP and vimentin while exhibiting classical radial glial morphology [36]. However, in those cultures, the con-fluent monolayer predominantly comprised thin, fibroblast-like cells aligned in parallel, interspersed with occasional islands of epithelioid cells. Within the core of radial glia colonies in our primary cultures, immunocytochemical staining for UCHL1 (also known as PGP9.5), a highly specific marker of neuronal lineage and structural integrity, confirmed the presence of substantial neuronal production. This neuronal marker belongs to the ubiquitin carboxy-terminal hydrolase (UCH) family of deubiquitinating enzymes, specifically used in neurobiology to demonstrate consistent neurogenesis [43]. The morphology of seabream neuroglial cell bodies in this model aligns with previous in vivo brain section observations evaluating domoic acid neurotoxicity, thereby validating our novel marine neuroglial system as a reliable platform for neurotoxicological research [44].

Although research remains limited, existing evidence demonstrates that *Sparus aurata* shares the high neurogenic and regenerative capacities observed in other major teleost models, such as the *Danio rerio* [33,45]. The presence of adult neural stem and progenitor cells (aNSPCs) and lifelong neurogenesis in the gilthead seabream has been documented in a limited number of in vivo studies, which are highly consistent with our current findings. Unlike zebrafish, *S. aurata* exhibits distinct slow- and fast-cycling NSPC populations, providing a robust platform to resolve neural stem cell kinetics. By overcoming the tissue mass limitations and technical scaling bottlenecks inherent to smaller model organisms, this marine teleost system offers a distinct advantage for advanced neurobiological applications and cell-based therapeutic screenings.

The high viability and long-term stability of these primary cultures validate *Sparus aurata* as a scalable, high-value cell source for biomedical applications and future tissue engineering. Elucidating the mechanisms of regenerative neurogenesis across different biological contexts is essential for repairing CNS lesions, reversing neurodegenerative processes, and developing effective therapeutic strategies for neural disorders in both teleosts and mammals. Our findings confirm that radial glia serve as the primary neural stem cell reservoir in the gilthead seabream brain, conferring exceptional structural and cellular plasticity to regenerating nervous tissue and establishing this marine species as a premier comparative model for adult neurogenesis, neuronal migration, and tissue repair.

In addition, data from gilthead seabream neurotoxicity assays underscore a historic reliance on in vivo and ex vivo exposure regimes, highlighting a distinct lack of validated in vitro screening systems for marine xenobiotics. This limitation is compounded by the dearth of Mediterranean fish cell lines suitable for examining viral neurosusceptibility and subsequent neurological damage [46]. Because various viral taxa cause extensive CNS inflammation and neuronal injury in fish, engineering novel in vitro models to investigate host pathogen dynamics is highly advantageous [46]. Ultimately, these systems allow investigators to precisely track viral evolution concurrently with host pathogenic responses, providing an indispensable framework for deciphering viral neuropathogenesis [47].

Undoubtedly, pioneering in vitro studies of this nature require suitable, readily available, and purified antibodies directed against specific fish protein markers. However, species-specific antibodies, particularly for seabream, targeting CNS cell differentiation markers remain scarce due to the limited number of suppliers catering to fish research. We therefore utilized commercial antibodies whose cross-reactivity with fish proteins has been documented in the literature. Because commercial antibodies are almost exclusively raised against mammalian antigens, success depends on selecting polyclonal antibodies or specific clones known to cross-react with teleosts [1]. In our previous research on *Sparus aurata*, we evaluated the potential cross-reactivity of commercial clones with target fish proteins through sequence homology analysis [25,27,48]. For instance, Alunni et al. [1] demonstrated that GFAP is highly conserved across evolution, confirming cross-reactivity with mammalian anti-GFAP. The antibodies used in this study (e.g., GFAP, NeuN, UCHL1, NF200, Vimentin, β-III-tubulin, CD44, MAP2, Nestin, PCNA) have been previously validated for teleost tissues or *Sparus aurata* primary cultures in published literature (e.g., neurofilament and GFAP in zebrafish embryo cultures by Ghosh et al. [49]; vimentin and GFAP immunoreactivities in the brain of gray mullet by Arochena et al. [50]; characterization of GFAP and astroglial architecture in the rainbow trout brain by Alunni et al. [1]; all aforementioned antibodies in seabream liver and brain primary cultures via immunocytochemistry or immunoblotting in our previous studies [25,27,48]). Ultimately, further advancements in marine biotechnology are required to expand the suite of molecular tools and markers available for primary marine cell cultures.

### Future Directions and Limitations

This study provides fundamental evidence demonstrating how a marine teleost model can facilitate advanced neurobiological applications, confirming the high viability of primary *Sparus aurata* neuroglial cells for long-term experimentation and clarifying the regenerative potential of radial glial stem cells. Evolving methodologies now enable the controlled and reproducible fabrication of bioengineered neural constructs designed to repair damaged nervous tissue. Recent ex vivo studies have leveraged neuroanatomical and functional data to guide neuroregenerative strategies using pre-engineered 3D neural constructs developed via neurosphere cultures or biohybridized microsystems [51]. By leveraging the developmental mechanisms underlying cell migration and axonal guidance, it is possible to create transplantable, microengineered neural constructs; these act as pro-regenerative living scaffolds, providing synergistic signals aimed at accelerating the functional reconnection of neural pathways compromised by disease or injury [51,52]. Furthermore, insights from scaffold-based designs have enabled the development of engineered neural networks that utilize physical microchannels to guide long-distance axonal growth [51]. Nevertheless, several significant limitations regarding future translational applications must be acknowledged. First, current bioengineering approaches rely on evolving methodologies for the controlled fabrication of engineered neural constructs; while these constructs are promising for repairing damaged nervous tissue or establishing predictive in vitro models, they currently serve more as tools for formulating research hypotheses than as definitive proof of therapeutic efficacy. Second, the absence of validated interspecies co-culture models limits our ability to replicate the host’s native microenvironment, representing a critical obstacle given the structural and functional complexity of nervous tissue. Finally, there remains a scarcity of mechanistic studies evaluating regenerative therapies based on the implantation of such neural networks.

Therefore, future research must utilize living neural scaffolds to develop transplantable, bioengineered neural networks while systematically mapping interspecies compatibility and functional recovery. Standardized longitudinal studies are required before transitioning from preclinical animal models to translational applications. Additionally, validating these cellular platforms will enable the development of high-potential cell products to evaluate therapeutic efficacy in neurodegenerative disease models. Ultimately, this specialized teleost model bridges the gap between basic neurobiology and applied bioengineering, providing a versatile platform for seeding next-generation 3D bioengineered scaffolds and interspecific biohybrid constructs [51]. Future investigations leveraging this system will be pivotal in unraveling the precise evolutionary and molecular mechanisms that govern constitutive CNS regeneration in teleosts.

## 5. Conclusions

In conclusion, this study establishes a well-characterized in vitro model of the gilthead seabream (*Sparus aurata*) CNS, demonstrating that its RG cells function as multipotent, self-renewing neural stem cells with high neuroregenerative potential. Our morphological and immunocytochemical profiling delineates the structural organization of teleost neurogenic niches and maps the pathways through which RG progenitors sustain the long-term production of neuronal and glial lineages. Furthermore, by tracking live nuclear dynamics and cellular translocation, we provide direct evidence of distinct, highly efficient modes of neuronal migration along primary radial scaffolds, a mechanism previously uncharacterized in seabream. The morphological shifts observed in these migrating neurons, including sequential nuclear dilation and elongation, represent a defining hallmark of teleost locomotion. The dynamic architecture of RG fibers serves as a structural scaffold that actively supports sustained neuronal growth, as evidenced by mitotic and translocating nuclei along the glial processes.

By serving as the primary reservoir of neural progenitors, these niche cells confer exceptional structural and cellular plasticity and regenerative capacity to the nervous tissue. From a comparative biology perspective, the consistent neurogenic activity and prolonged proliferative dynamics observed in *Sparus aurata* primary cultures reflect the extraordinary neural plasticity inherent to the adult teleost brain. Consequently, this model represents a pivotal comparative framework for CNS repair. Deciphering these conserved cellular pathways offers actionable evolutionary insights that may guide the development of innovative, translationally viable neural repair strategies for the brains of higher vertebrates. Furthermore, by faithfully replicating the teleost CNS in vivo environment, this alternative in vitro platform offers an immediate and accessible resource for screening applications in pharmacotoxicology, physiology, virology, neuropathology, and marine neuroimmunology, fields that remain under-investigated.

## Figures and Tables

**Figure 1:**
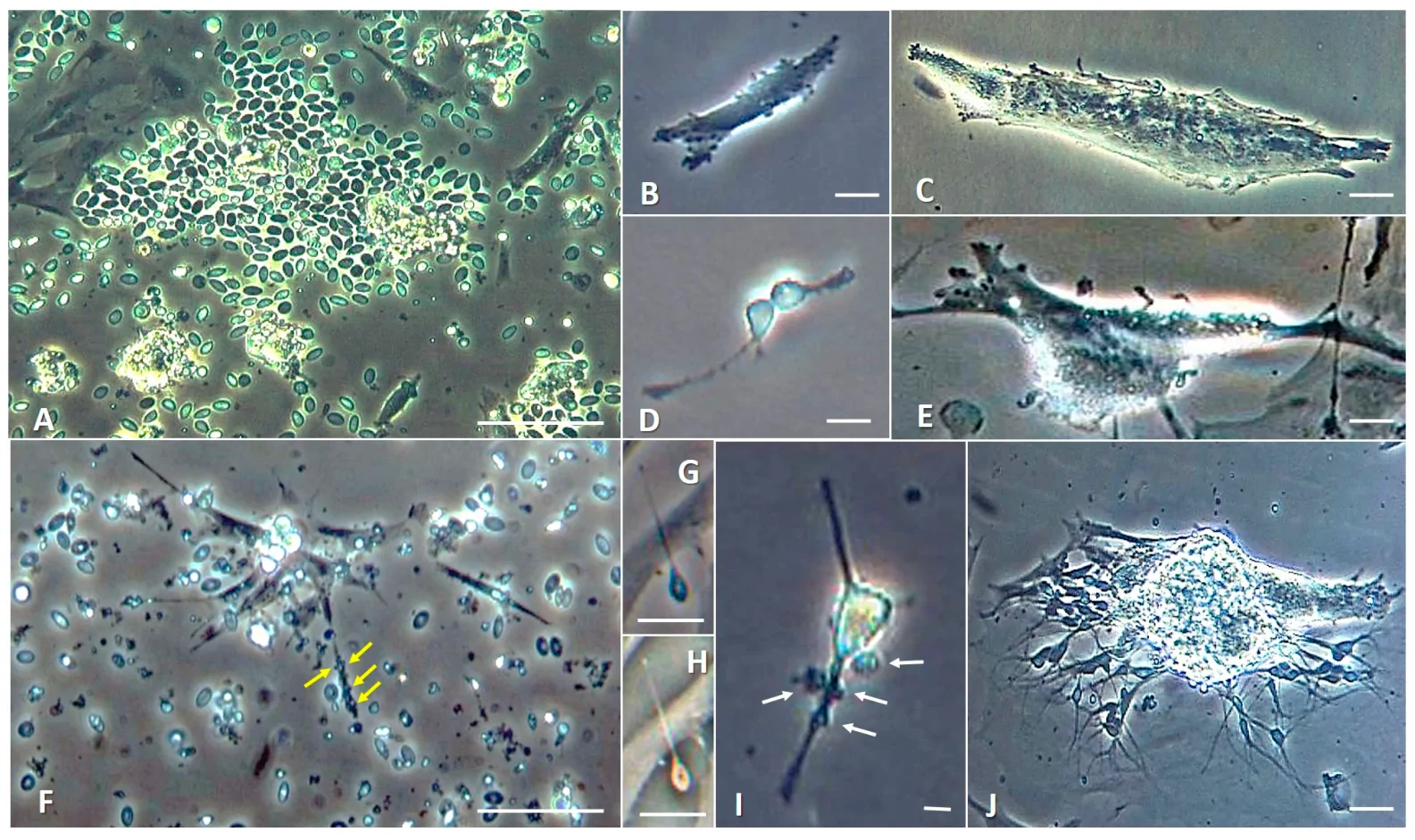
Morphological development of *Sparus aurata* primary neuroglial cells in vitro. Representative phase-contrast images showing: (**A**) Freshly isolated neural stem cells (NSCs) at 1 day in vitro (200×). (**B**) Radial glial stem progenitors (RGSPs) cluster at 2 days in vitro (400×). (**C**,**D**) Elongated attached NSCs at 2 days in vitro (200×). (**E**) Elongated attached NSCs at 4 days in vitro (200×). (**F**) Neuron progenitor cells (NPCs) colony at 6 days in vitro with dividing neuroblasts (yellow arrows) (200×). (**G**) Elongated NPC at 2 days in vitro and inverted view (**H**) (200×). (**I**) Tripolar NSC with dividing neuroblasts (white arrows) (200×). (**J**) Highly branched radial glial cells (RGCs) at 4 days in vitro (200×). Scale bar = 50 μm (**all**); 10 μm (**I**).

**Figure 2:**
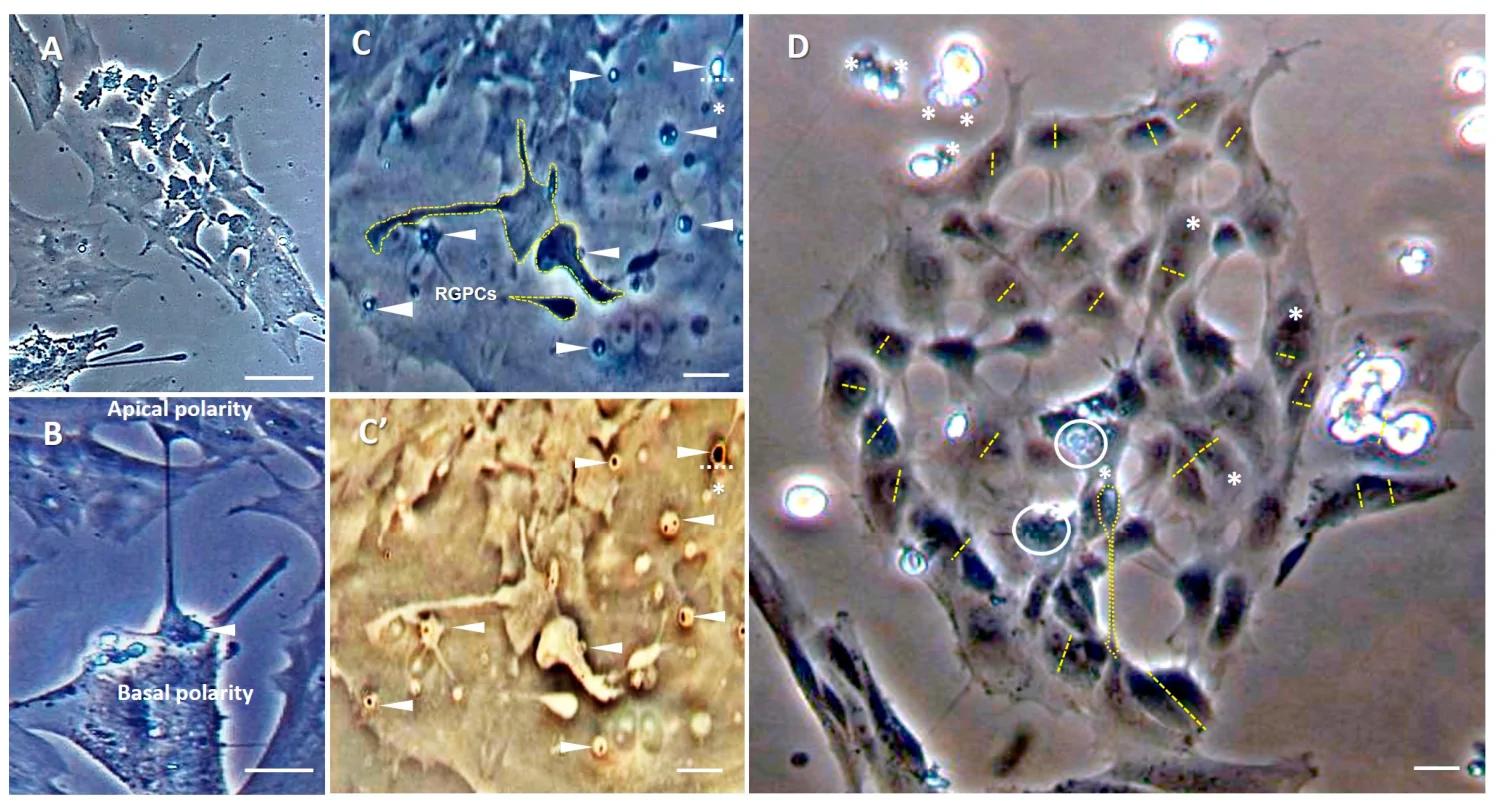
Morphological characterization of RGSPs forming colonies in *Sparus aurata* primary cultures. Phase-contrast micrographs showing the emergence and elongation of radial processes during the first few days in vitro. (**A**) RGSPs initiating process extension. (**B**) Basal cones generating long directional extensions and lateral processes. Glial endfeet help define the apical–basal polarity of the cell, regulate basal process elongation, and form the scaffold essential for proper neuronal migration. The formation of primary extending processes represents a crucial event in establishing the polarized radial glial scaffold (200×). (**C**,**C’**) High-magnification views of multipotent radial glial stem colonies showing prominent growth cones; white arrows indicate newborn glial stem progenitors, the white dashed line indicates the plane of division of the newly generated cell (400×). (**D**) Basal radial glial cells undergoing rapid division within the colony. White asterisks indicate early basal cells, white arrows indicate developing stem cells, and the white circle delineates an actively proliferating stem cell niche. Asymmetric division generates one apical and one basal progenitor, whereas vertical cleavage planes promote self-renewal. Dashed yellow outlines highlight numerous symmetric divisions of bRGSP cells within the colony (400×). RGPCs: Radial Glial Progenitor Cells. Scale bar = 50 μm (**all**).

**Figure 3:**
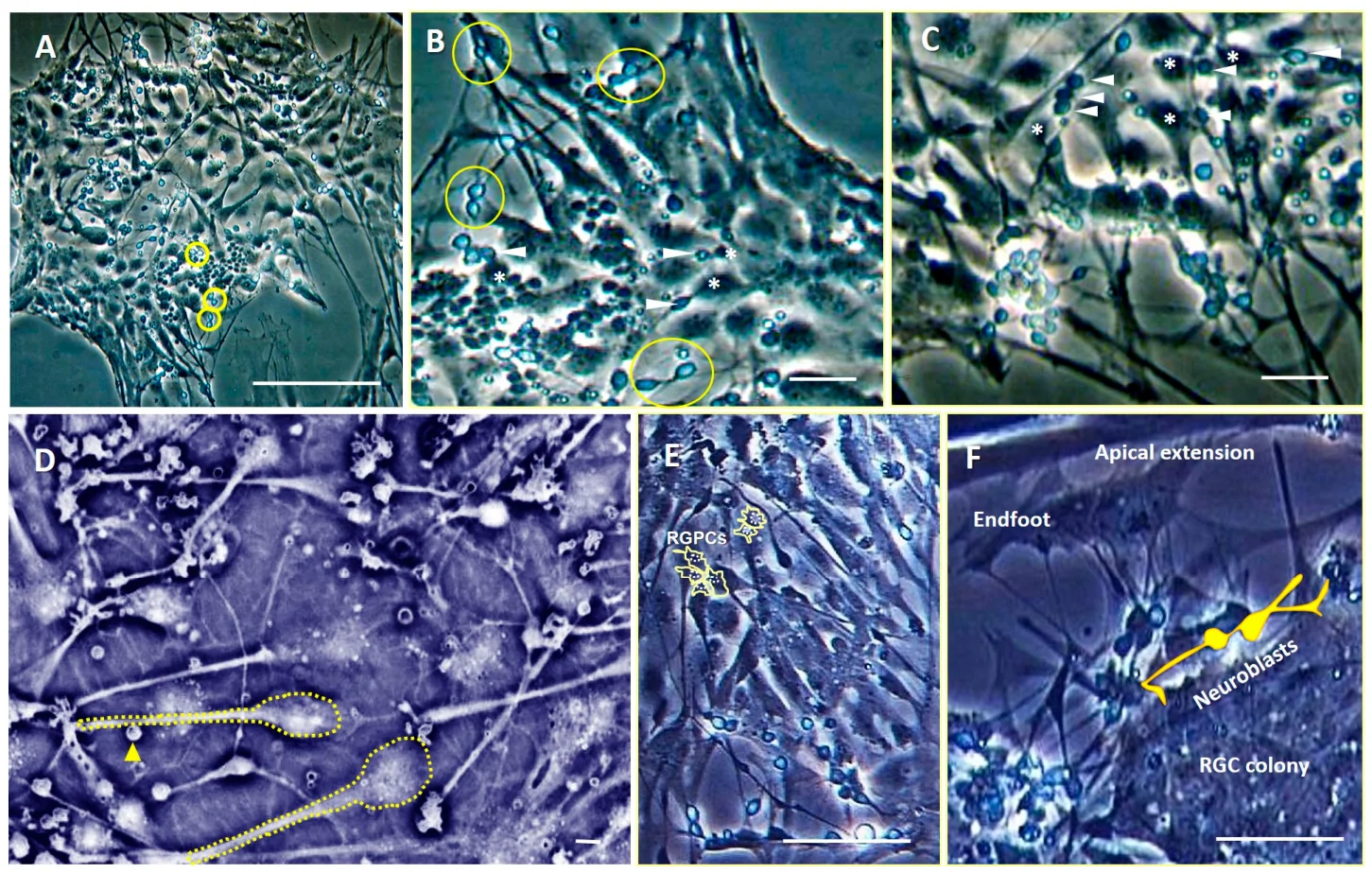
Differentiation of radial glial colonies during early neurogenesis. Phase-contrast micrographs of *Sparus aurata* neuroglial cultures illustrating radial glia progeny derived from neural stem cells. (**A**) Morphology of a radial glial colony after 2 weeks in vitro, showing enrichment of dividing neurons (yellow circles; 200×). (**B**) Magnified and 180-degree rotated view of the central area in (**A**), highlighting granular neuronal production (yellow circles), paired newly formed neurons along the fiber, and the initial emerging neuron (white arrowhead) from the basal cone (white asterisk). (**C**) Multipotent radial glial colony displaying growth cone emergence sites along radial processes (asterisks, white arrows) and active neuronal proliferation zones (white arrows) after 2 weeks of culture (400×). (**D**) Inverted phase-contrast view of the asterisk-marked cells in (**C**), with growth cone borders delineated by yellow dashed lines and a newborn neuroblast indicated by a yellow arrow (200×). (**E**) Radial glial progenitor cells extending processes and proliferating in differentiation-enriched medium (200×). (**F**) Early-stage neurogenesis driven by basal radial glial progenitors, with neuroblasts (yellow outlines) migrating transversely along the colony periphery (200×). Scale bar = 50 μm (**all**); 10 μm (**D**).

**Figure 4:**
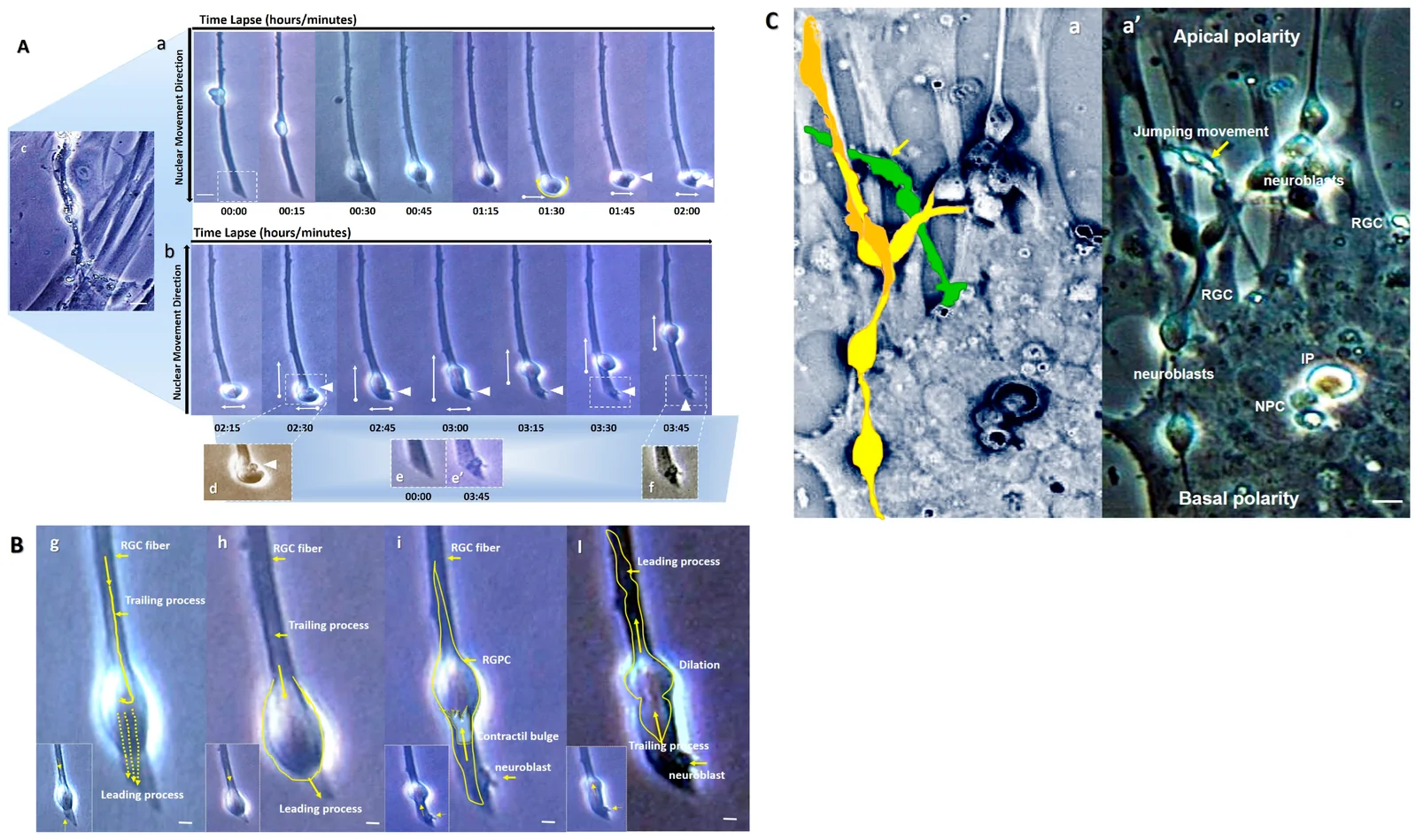
Radial nucleokinesis and neurogenic migration along radial glial cell fibers in *Sparus aurata* primary neuroglial cultures. (**A**) *Mitotic somal translocation (MST) and neurogenesis.* Sequential phase-contrast time-lapse micrographs, illustrating real-time MST and nucleokinesis in a basal radial glial progenitor cell (bRGPC) along glial fibers in a 2-week-old culture. Images were captured every 15 min over a 12 h total duration at 18 °C and low CO_2_ system in L-15 medium. (**a**) bRGPC undergoing MST, with the nucleus translocating toward the distal terminus of the glial fiber prior to division. Time is in hours:minutes. (**b**) Post-mitotic nucleus resuming upward nucleokinesis along the fiber, while the newly formed daughter cell establishes adhesions surrounding the parental scaffold. (**c**) High-magnification views of the boxed regions in (**a**,**b**); white arrows in (**a**) highlight membrane rearrangement bubbles, and white arrows in (**b**) indicate a mitotic bulge and newly generated neuroblast. (**d**–**f**) Inverted high-magnification views of boxed regions; white arrowheads mark the apical division process and nascent cellular bud formation (200×). Scale bar = 50 μm (all). (**B**) MST and neuronal locomotion mechanics during neurogenesis. Selected high-magnification phase-contrast micrographs from panel (**A**) illustrating bidirectional migratory dynamics along radial fibers. (**g**) Nucleus translocating toward the distal terminus prior to division; yellow dashed lines indicate microtubule tracks bridging the nucleus and apical fiber. (**h**) Nucleo-centrosomal coupling extending proximal–distally before mitosis halts nucleokinesis, yielding an adherent neuroblast. (**i**) Parental progenitor nucleus retracting and migrating back up the fiber, accompanied by a posterior contractile bulge. (**l**) Parental progenitor migrating toward its origin site while the newborn neuroblast extends processes around the parental fiber. Solid yellow lines track the migratory paths of the nucleus and soma (200×). Scale bar = 10 μm. (**C**) Tangential neuronal migration along RGC fibers. (**a**,**a’**) Phase-contrast micrographs showing tangential migration across glial scaffolds and active neurogenesis. (**a**) Inverted phase-contrast view of (**a’**) with migrating neuroblasts false-colored (yellow, orange, green) to highlight transverse migratory trajectories and complex fiber–axon interlace (400×). Tangential migration occurs transversely across adjacent fibers rather than parallel along the primary longitudinal axis. IP: Intermediate progenitor; NPC: Neuronal progenitor cell; RGC: Radial glial cell. Scale bar = 50 μm (**all**).

**Figure 5:**
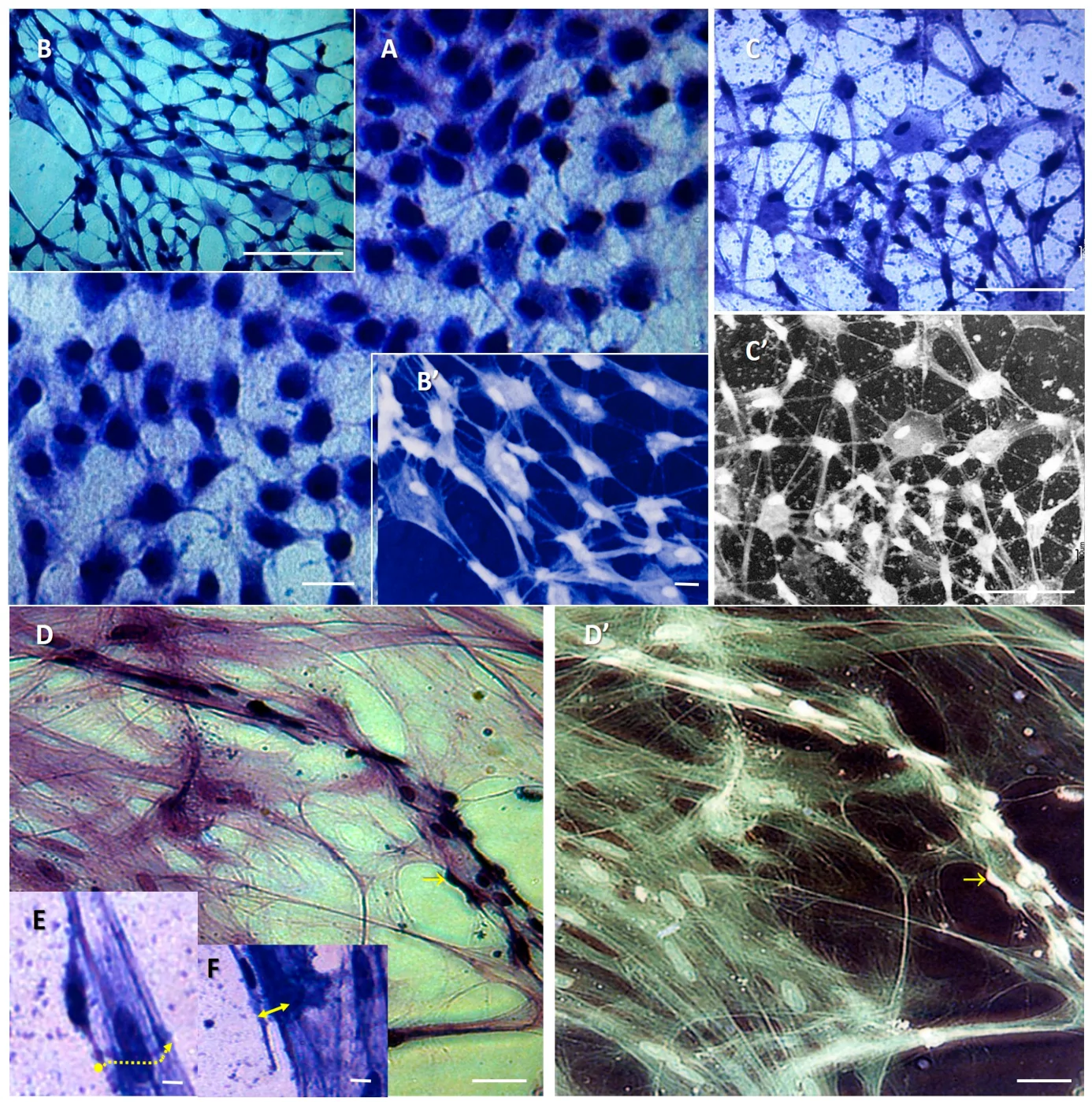
Scaffolding formation in *Sparus aurata* neuroglial cultures after 2 weeks. (**A**–**D**) In differentiation-enriched medium, primary neuroglial cultures develop an astrocytic monolayer supporting a dense, interlacing neuroblast network that creates a cooperative scaffold. (**D’**) Inverted-negative microscopic view of (**D**), showing neurons and nuclei in white. (**D**,**D’**) Adherent neuroblasts are indicated by yellow arrows (**E**) Representative micrographs, enlargedview, of neuroblasts climbing a glial fiber (dashed lines with yellow arrows). (**F**) Close morphological interaction between neuroblasts and radial glial processes, yellow lines and arrows highlight neuronal processes embracing the fiber, with specific membrane adhesion areas. Scale bar = 50 μm (**all**); 10 μm (**B’**,**E**,**F**).

**Figure 6:**
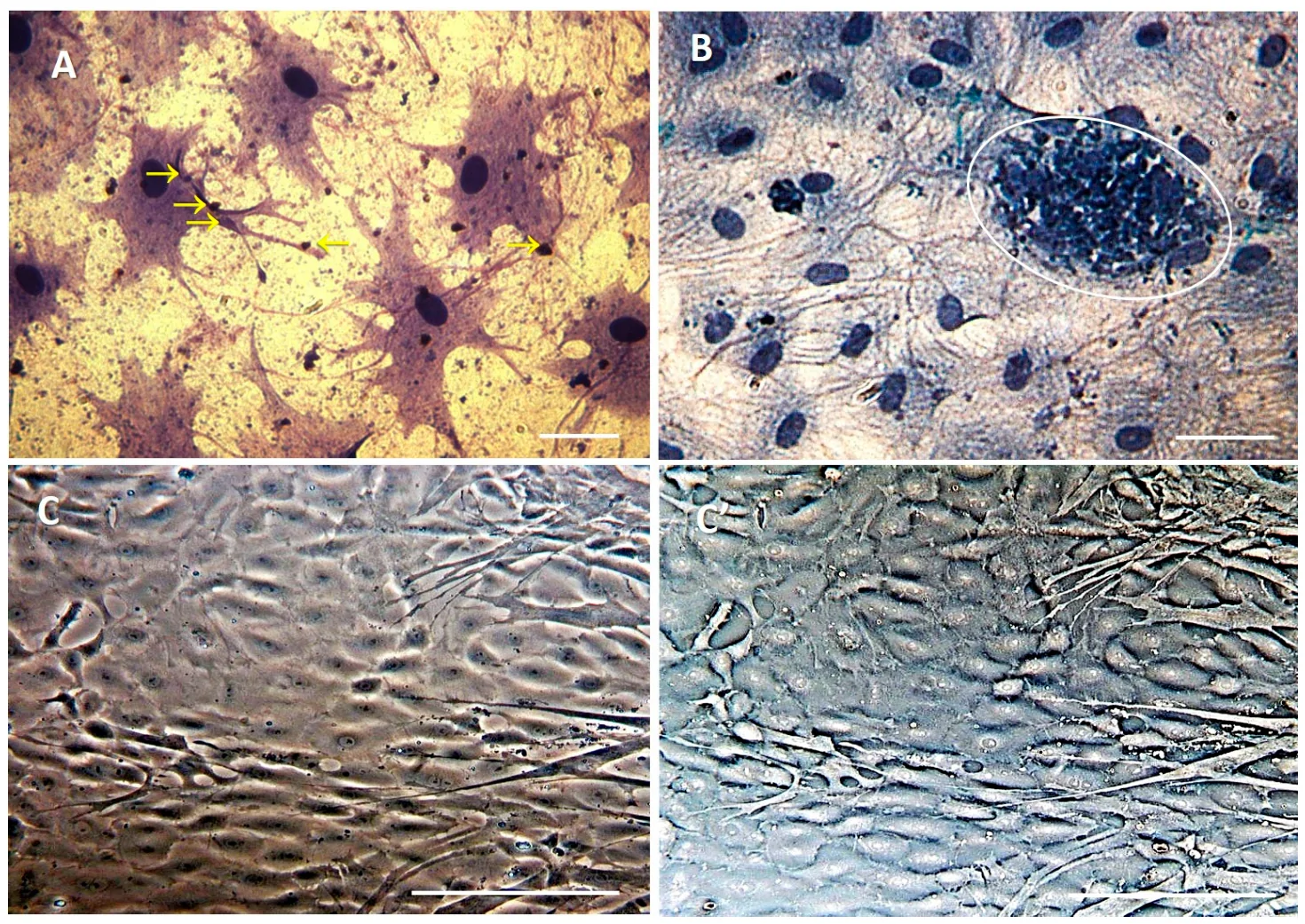
In vitro differentiation, cellular morphologies, and niche formation in *Sparus aurata* neuroglial cultures. (**A**) Primary neuroglial cultures in enriched medium showing differentiation into type-II astrocytes with highly branched, stellate, and neuron-like morphologies (yellow arrows) derived from neural progenitor cells in 1-week-old cultures. (**B**) Radial glia colonies and neural niche formation in 3-week-old cultures, with highly proliferative neurogenic niches delineated by white circles (400×). (**C**,**C’**) Primary neuroglial cultures differentiating into mature astrocytes that form a cohesive monolayer, accompanied by neuronal generation in 2-week-old cultures (200×). Scale bar = 50 μm (**all**).

**Figure 7:**
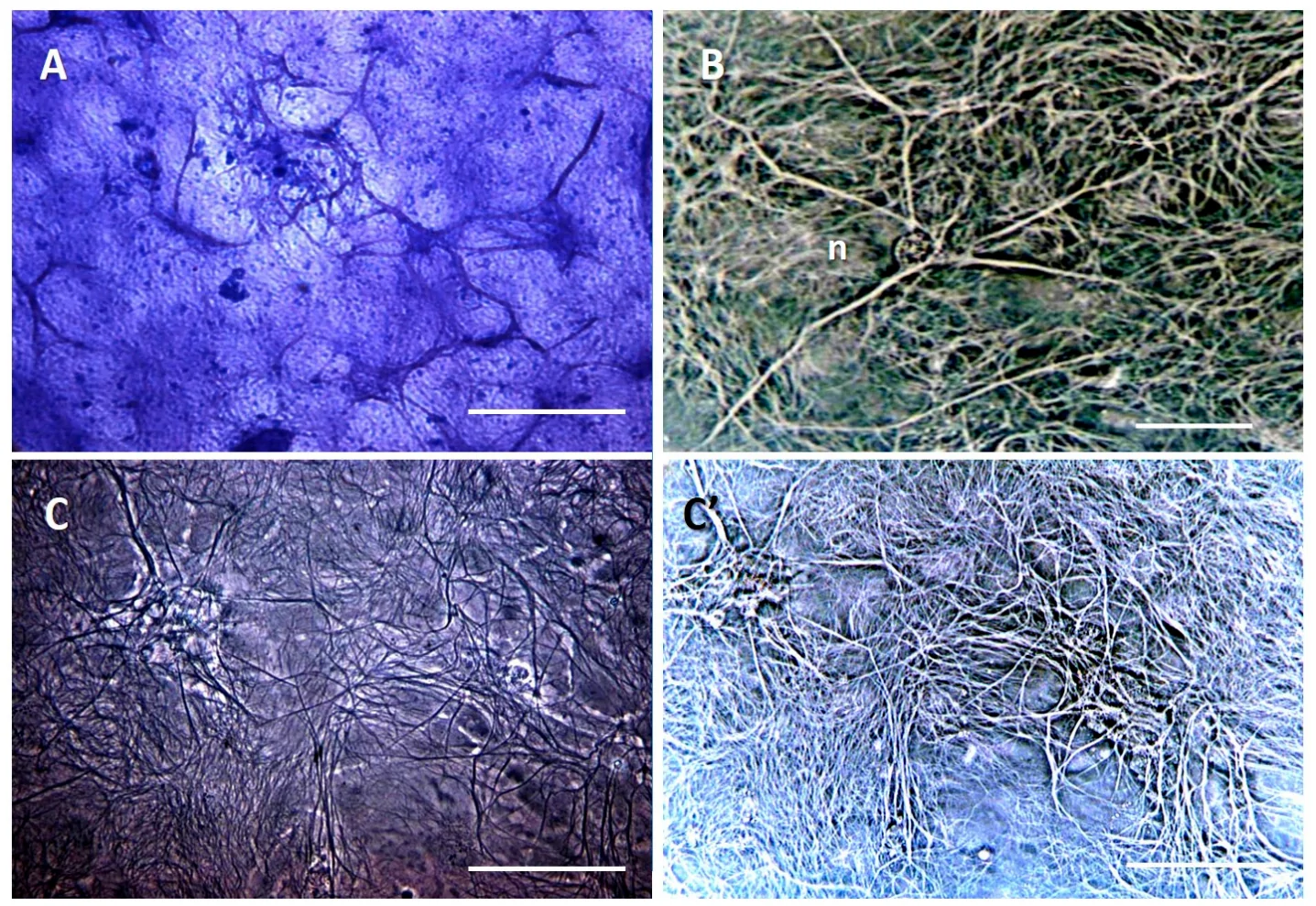
Radial glial progenitors generate neurons and astrocytes in vitro in long term cultures. (**A**) Astrocytic monolayer displaying dense process branching interspersed with cells with neuronal-like morphology and elongating neurite like processes. (**B**) Neuronal generation in long-term cultures, where “n” indicates a neuronal-like morphology exhibiting highly arborized axonal and dendritic like processes overlying the astrocytic monolayer. (**C**) Extensive coverage of the monolayer by dense dendritic and neuritic networks alongside well-defined neuronal somata by 4 weeks in culture, with neurite-like arborization and length peaking at 2 months in vitro. (**C’**) Inverted phase-contrast view of (**C**), highlighting the intricate convolution of neurites surrounding a neurogenic niche. (**A**,**C**,**C’**) (200×); (**B**) (400×). Scale bar = 50 μm (**all**).

**Figure 8:**
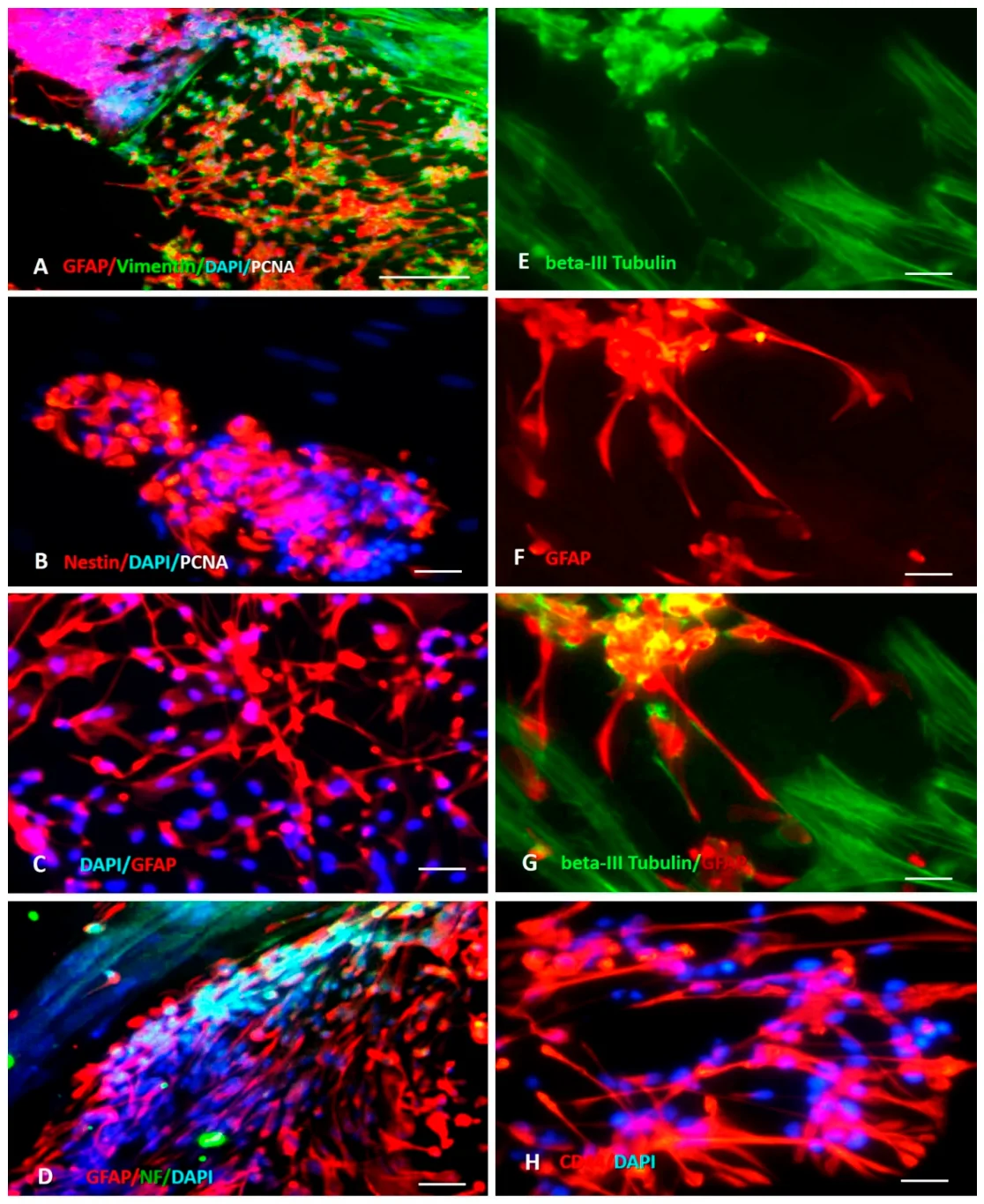
Immature and mature radial glia colonies and differentiated astrocytes. Images display the expression of specific markers using double immunofluorescence. (**A**) Radial glial cells (RGCs) within large colonies maintained elongated, spindle-like processes, exhibiting intense staining for both Vimentin^+^ (green) and GFAP^+^ (red) after 3 weeks in vitro; merged images. (**B**) Prominent Nestin^+^ expression (red) within the inner core of proliferating RGC colonies, confirming a progenitor phenotype. (**C**) Mature RGCs immunostained for GFAP^+^ (red) showing positive staining in cell bodies and filopodia, with nuclei counterstained with DAPI (blue). (**D**) GFAP^+^ mature astrocytes (red) interacting with Neurofilament^+^ (NF^+^; green) axons after 1 month in vitro, with DAPI labelled nuclei (blue). (**E**–**G**) GFAP^+^ (**F**) after 1 month in vitro, with astrocytes at the outer periphery featuring elongated processes aligned with associated β-III-tubulin^+^ neuronal fibers (green) ((**E**,**G**) merged). (**H**) Differentiated scaffolding astrocytes expressing CD44^+^ at 1 month of culture in vitro. Scale bar = 50 μm (**all**). DAPI: 4′,6-diamidino-2-phenylindole; GFAP: Glial fibrillary acidic protein; RGCs: radial glial cells; NF: Neurofilament; PCNA: Proliferating cell nuclear antigen.

**Figure 9:**
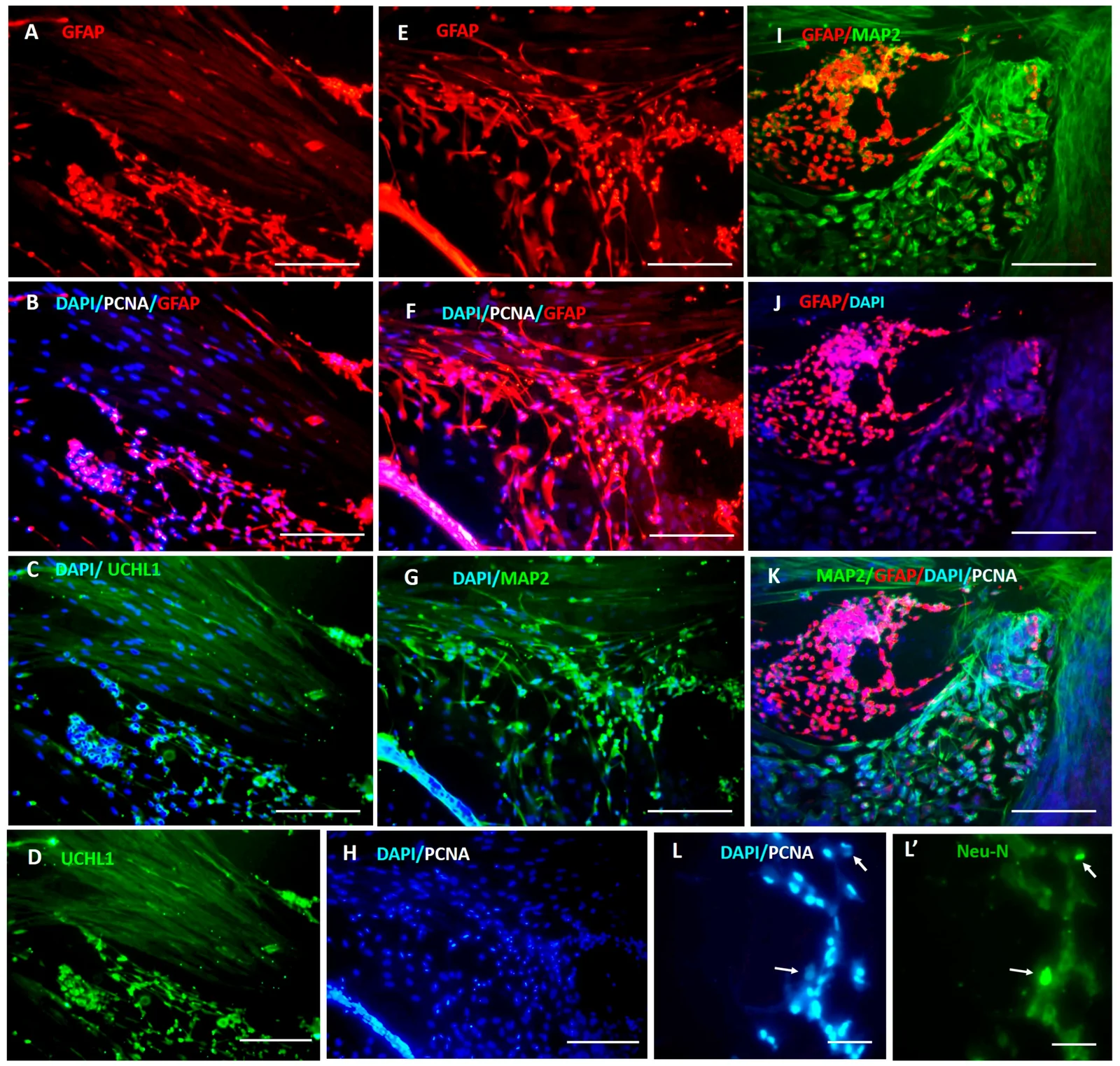
Colocalization of neurons and astrocytes within radial glial colonies at 2 months in vitro. Images display the expression of specific markers using double immunofluorescence. (**A**–**D**) Actively replicating neuronal progenitors (UCHL1^+^/PCNA^+^) with DAPI counterstain (blue) within the center of a GFAP^+^ RG colony. (**E**–**H**) Characterization of the RGC scaffold, GFAP^+^ (red) and MAP2^+^ (green) immunostaining, showing proliferative cell clusters exhibiting neurogenic activity (MAP2^+^/PCNA^+^) with nuclei DAPI counterstained (blue) along the fibers; a prominent complex of RG fibers (GFAP+) with numerous adherent neurons (MAP2+) that are replicating (PCNA+) is visible at the bottom left. (**I**–**K**) Outer boundary of the colony displaying differentiated astrocytes with GFAP^+^ filopodia and mature neurons with extended processes, while the inner zone (**J**,**K**) contains mitotic radial glia clones (GFAP^+^/PCNA^+^) with DAPI counterstain. (**L**,**L’**) Differentiating neurons (NeuN^+^) progressively cease dividing (PCNA^−^) and acquire the marker profile of post-mitotic neurons (indicated by white arrows), surrounded by actively proliferating progenitor cells (NeuN^−^/PCNA^+^). Scale bar = 50 μm (**all**). DAPI: 4′,6-diamidino-2-phenylindole; GFAP: Glial fibrillary acidic protein; MAP2: Microtubule-associated protein 2; Neu-N: Neuronal nuclei; PCNA: Proliferating cell nuclear antigen; UCHL1: Ubiquitin C-terminal hydrolase L1.

**Figure 10:**
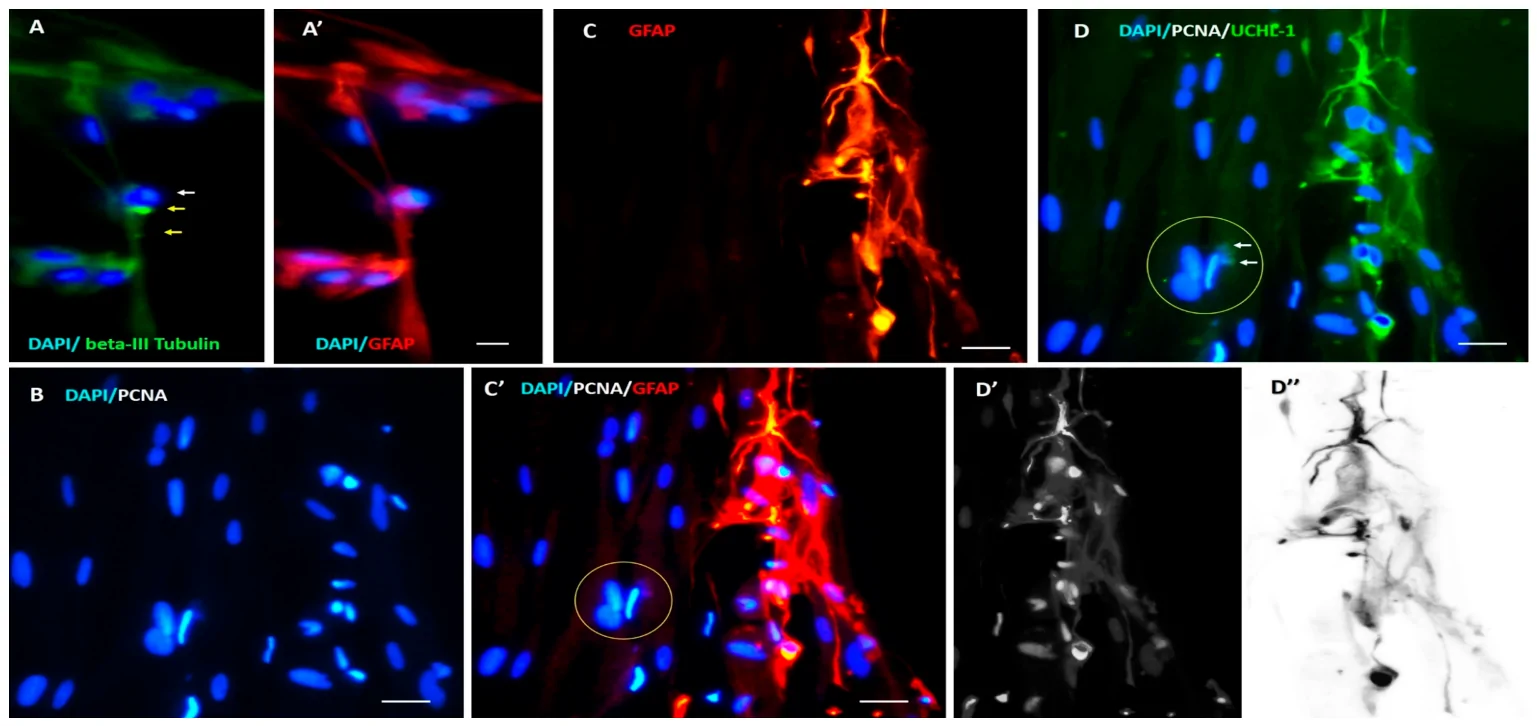
Tangential and radial neuronal migration and structural interactions along radial glial cell fibers. Images display the expression of specific markers using double immunofluorescence. (**A**,**A’**) Neurons (ß-III-tubulin+, green) aligned with a radial glial fiber (GFAP+, red) at 2 months in vitro with DAPI counterstain (blue), white arrowhead indicates the neuronal soma with a prominent cytoplasmic bulge (green), and the ß-III-tubulin+ signal highlights the perinuclear ring and centrosome/MTOC (yellow arrowheads). (**B**–**D**) Elongated nuclei directed into neurites (green) during tangential and radial migration, showing GFAP+ areas (red) (**C**,**C’**) combined with UCHL1+ neuronal clusters (**D**) and (**B**,**C’**,**D**) PCNA+/DAPI+ nuclei (white/blue), reveals the proliferative capacity of cells, as demonstrated by the labeling of mitotic nuclei stained with anti-PCNA Ab and counterstained with DAPI (blue). Asymmetric nuclear divisions and nuclear elongation during translocation (yellow circles) are observed. (**D**) Neurons with positive immunoreactivity to UCHL1 marker; white arrows indicate specific interactions that facilitate neuroblast attachment to radial glial cells along structural radial fibers, during nuclei elongation (200×). (**D’**,**D’’**) High-contrast images showing the detailed organization of the branched process structures against black-and-white backgrounds. (**D’**) represents a negative image showing the bright white branches of the neuroglial tree against a deep black field, while (**D’’**) represents a positive image showing dark cellular extensions and branched networks against a white field. Scale bar = 50 μm (**all**).

**Figure 11:**
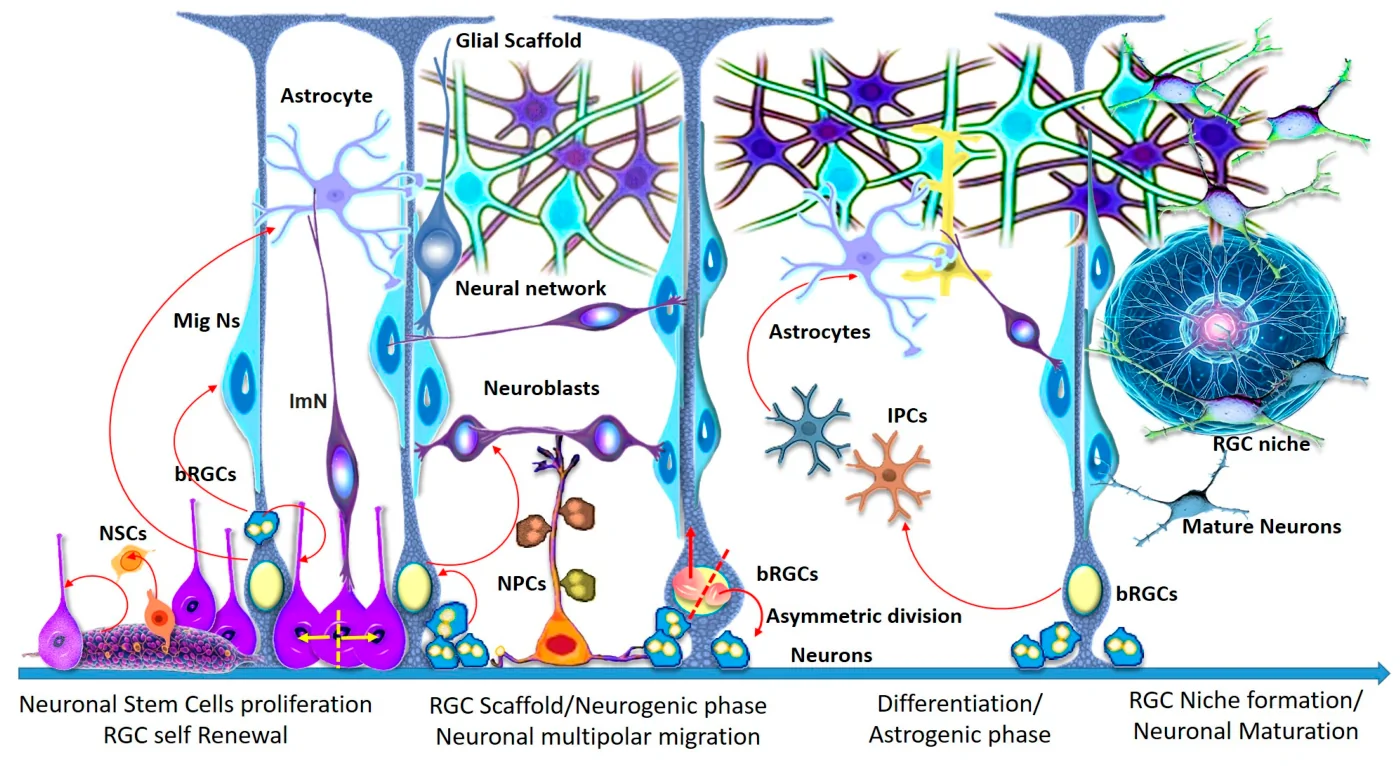
Schematic overview of early-stage in vitro neurogenesis, development, and differentiation in *Sparus aurata* neuroglial cultures. The drawing illustrates the in vitro division patterns and emerging cell types identified throughout *Sparus aurata* neurogenesis in this study. Radial glial progenitor cells (RGCs) divide asymmetrically to generate an apical progenitor and a basal progenitor, with the latter driving radial colony expansion. Most basal progenitor divisions occur vertically, promoting colony self-renewal (yellow dashed line and yellow arrows). Early in development, RGCs divide symmetrically to yield two identical RGCs, expanding the initial progenitor pool. During intermediate differentiation, asymmetric neurogenic division occurs: one daughter cell remains an RGC to maintain the progenitor pool, while the other becomes a mature neuron or intermediate progenitor cell (IPC). In late development, RGCs undergo a final symmetric gliogenic division where both daughter cells differentiate into glial lineages, such as astrocytes, thereby depleting the embryonic RGC pool. Cleavage plane orientation directs cell fate: vertical cleavage typically leads to symmetric division, whereas horizontal or oblique cleavage triggers asymmetric division via uneven distribution of cellular components. Neural stem cells (NSCs) utilize analogous mitotic mechanisms: symmetric self-renewal produces two identical multipotent NSCs to expand or restore the stem cell pool, whereas asymmetric self-renewal yields one NSC and one committed progenitor cell within neurogenic niches.

## Data Availability

The raw data supporting the conclusions of this article will be made available by the authors on request. The datasets are not publicly available because they contain unpublished data that form part of ongoing research and future planned analyses.
